# Supplementary material for: Exploring the use of a gamified intervention for encouraging physical activity in adolescents: a qualitative longitudinal study in Northern Ireland
Source: BMJ Open. 2018 Apr 20;8(4):e019663. doi: 10.1136/bmjopen-2017-019663 (PMC5914771; doi:10.1136/bmjopen-2017-019663)
Supplement: Supplementary file 1 [file bmjopen-2017-019663supp001.pdf]

Supplementary Material I: Topic Guide

Topic Guide			
**Pre-intervention**	**End of phase one (External regulation; Introjected regulation)**	**End of phase two (Introjected regulation; Identified regulation)**	**12 months post-baseline follow up (Integrated motivation)**
**Explore the barriers and facilitators to PA**<br><br>What stops you from being physically active? (Relational support)<br><br>Role of parents/friends in your PA? (Autonomy support; Relational support)<br><br>Any ideas to increase PA? (Autonomy support)<br><br>Opportunities for PA within school/community? (Autonomy support; Relational support)	**Explore the experiences of StepSmart**<br><br>How easy is it to find ways in which to be more active? (Autonomy support)<br><br>Did completing the StepSmart Challenge with friends make it easier? Or more pressure? (Autonomy support; Relational support)<br><br>Do you enjoy telling people about your success in terms of PA (e.g. showing others your steps)? (Perceived competence; Relatedness)	**Explore the how participants felt about the different competition elements**<br><br>How did the team competition compare to the individual competition? (Relational support)<br><br>Some of the class were more/less active than you. How did you feel when you compared your steps to the class? (Perceived competence)<br><br>Did team members encourage you to be active or not? (Autonomy support)<br><br>How did you feel if you didn't contribute to the team/school's step count? (Perceived competence)	**Explore if there has been a change in PA (formation of habit)**<br><br>One year on: how active are you now (compared to before you took part)?<br><br>Did you make changes to your PA? Why/why not? *(changes to routine ... active travel, walking with friends etc.)*<br><br>If you did make any changes, which are you still doing? What strategies helped? (Autonomy support)
**Explore the acceptability of the intervention components**<br><br>Have you ever heard of…or used a pedometer?<br>What is it you (dis)like about them?<br><br>Feelings on writing down your own daily steps goals in a workbook.	**Explore the attitude towards PA**<br><br>Do you enjoy PA?<br><br>What are your main reasons for being active? (i.e. role of the prizes and other elements?)	**Explore how the StepSmart Challenge instigated any changes in participants PA**<br><br>How do you feel about PA since the StepSmart Challenge?<br><br>What have you enjoyed?	**Explore how participants felt about the StepSmart Challenge**<br><br>Why did you choose to participate?<br><br>Good/not so good aspects of the competition?<br><br>Did the competition motivate – no/at beginning/all throughout?

(Perceived competence; Autonomy support)<br><br>What features of a website would you find appealing?<br><br>How do you feel about entering a school-based competition?<br><br>Any advantages/disadvantages to team vs individual competitions<br><br>What prizes would motivate young people to take part?	Do you enjoy finding new ways of becoming more active?<br>(Autonomy support)<br><br>Do you think your motivation towards PA has changed?	Has the StepSmart Challenge increased your PA? If not, what would motivate you to be more active?<br><br>Since the StepSmart Challenge, what new opportunities have you found to be active?<br>(Autonomy support)<br><br>Anything outside your control stopping you from being active? (e.g. park proximity, family, other)<br>(Autonomy support; Relational support)	
	**To explore the consequences of PA**<br><br>Do you find yourself having more energy?<br><br>Do you feel more confident in being able to achieve things?<br>(Perceived competence)<br><br>How do you feel after you've completed some PA?<br><br>Do you think you are more active now than when you started? Why?	**Explore how participants felt about the use of the pedometer**<br><br>How often did you use the pedometer? What did you like/dislike about it? (e.g. Feedback)<br>(Perceived competence; Autonomy support)<br><br>How many of you are still using the pedometer? Why?	**Explore how participants felt about the team/individual competition**<br><br>Class vs school, was one enjoyed more?<br>(Autonomy support; Relational support)<br><br>Influence of friends on being active?<br>(Autonomy support; Relational support)<br><br>Opportunity to make new friends?<br>(Relational support)<br><br>Feelings about not contributing to the team/school step count?<br>(Perceived competence)<br><br>More motivated if friends wanted to do well in the competition?<br>(Relational support)

		**Explore how participants felt about the rewards/prizes**<br><br>What did you think of the prizes?<br><br>Tell me how well they worked to motivate you?<br><br>How did you feel if you didn't win a prize?<br>(Perceived competence)<br><br>If no prizes, but still a competition, would you still be as active? Why?	**Explore how participants felt about the rewards/prizes**<br><br>Type of prizes preferred, including ttrophy or a certificate?<br><br>Not competing against others, but beating your own goal?<br>(Autonomy support; Perceived competence)
		**Explore how the website and Facebook group were used**<br><br>Things you liked/didn't like about the website?<br><br>What did you think of Facebook group?<br>(Autonomy support; Relational support)<br><br>Which way would you prefer to get your information from?<br>(Autonomy support)	**Mood/motivation did these change at different stages of the intervention?**<br><br>Feelings if didn't make as many steps as others? Did that affect your mood?<br>(Perceived competence; Relational support)<br><br>If you did/didn't do well - did that motivate/demotivate you?<br>(Perceived competence; Relational support)<br><br>If felt like don't have a good chance to win would that make you stop trying?<br>(Perceived competence; Relational support)

Red text illustrates how items in the topic guide are linked the concepts of self-determination theory
