# Supplementary material for: Exploring the use of a gamified intervention for encouraging physical activity in adolescents: a qualitative longitudinal study in Northern Ireland
Source: BMJ Open. 2018 Apr 20;8(4):e019663. doi: 10.1136/bmjopen-2017-019663 (PMC5914771; doi:10.1136/bmjopen-2017-019663)
Supplement: Author's manuscript [file bmjopen-2017-019663.draft_revisions.pdf]

BMJ Open

BMJ Open is committed to open peer review. As part of this commitment we make the peer review history of every article we publish publicly available.

When an article is published we post the peer reviewers' comments and the authors' responses online. We also post the versions of the paper that were used during peer review. These are the versions that the peer review comments apply to.

The versions of the paper that follow are the versions that were submitted during the peer review process. They are not the versions of record or the final published versions. They should not be cited or distributed as the published version of this manuscript.

BMJ Open is an open access journal and the full, final, typeset and author-corrected version of record of the manuscript is available on our site with no access controls, subscription charges or pay-per-view fees (http://bmjopen.bmj.com).

If you have any questions on BMJ Open's open peer review process please email info.bmjopen@bmj.com

BMJ Open

# Exploring the use of gamification for encouraging physical activity in adolescents: A qualitative longitudinal study

| | |
|---|---|
| Journal: | *BMJ Open* |
| Manuscript ID | bmjopen-2017-019663 |
| Article Type: | Research |
| Date Submitted by the Author: | 19-Sep-2017 |
| Complete List of Authors: | Corepal, Rekesh; Queen's University Belfast, Centre for Public Health<br>Best, Paul; Queen's University Belfast, Centre for Evidence and Social Innovation<br>O'Neill, Roisin; Queen's University Belfast<br>Tully, Mark; Queen's University Belfast,<br>Edwards, Mark; University of Bristol, Centre for Exercise Nutrition and Health Sciences<br>Jago, Russ; University of Bristol, Centre for Exercise, Nutrition and Health Sciences<br>Miller, Sarah; Queen's University Belfast, Centre for Evidence and Social Innovation<br>Kee, Frank; Queen's University Belfast, UKCRC Centre of Excellence for Public Health (NI)<br>Hunter, Ruth; Queen's University Belfast, Centre for Public Health |
| <b>Primary Subject Heading</b>: | Qualitative research |
| Secondary Subject Heading: | Public health |
| Keywords: | PREVENTIVE MEDICINE, PUBLIC HEALTH, QUALITATIVE RESEARCH |

SCHOLARONE™
Manuscripts

# Exploring the use of gamification for encouraging physical activity in adolescents: A qualitative longitudinal study

Rekesh Corepal, Paul Best, Roisin O'Neill, Mark A. Tully, Mark Edwards, Russell Jago, Sarah Miller, Frank Kee, Ruth F. Hunter

Rekesh Corepal, UKCRC Centre of Excellence for Public Health/Centre for Public Health, Queen's University Belfast, Belfast, Northern Ireland

Paul Best, Centre of Excellence and Social Innovation, School of Social Sciences, Education and Social Work, Queen's University Belfast, Belfast, Northern Ireland

Roisin O'Neill, UKCRC Centre of Excellence for Public Health/Centre for Public Health, Queen's University Belfast, Belfast, Northern Ireland

Mark A. Tully, UKCRC Centre of Excellence for Public Health/Centre for Public Health, Queen's University Belfast, Belfast, Northern Ireland

Mark Edwards, Centre for Exercise Nutrition and Health Sciences, School for Policy Studies, 8 Priory Road, University of Bristol, Bristol, England

Russell Jago, Centre for Exercise Nutrition and Health Sciences, School for Policy Studies, 8 Priory Road, University of Bristol, Bristol, England

Sarah Miller, Centre for Evidence and Social Innovation, School of Social Sciences, Education and Social Work, Queen's University Belfast, Belfast, Northern Ireland

Frank Kee, UKCRC Centre of Excellence for Public Health/Centre for Public Health, Queen's University Belfast, Belfast, Northern Ireland

Ruth F. Hunter, UKCRC Centre of Excellence for Public Health/Centre for Public Health, Queen's University Belfast, Belfast, Northern Ireland

**Corresponding author:** Dr Ruth Hunter, UKCRC Centre of Excellence for Public Health/Centre for Public Health, Institute of Clinical Sciences B, Queen's University Belfast, Belfast, Northern Ireland, BT12 6BJ; E-mail: ruth.hunter@qub.ac.uk; Telephone: +44 (028) 90 978944

**Word count:** 4799

For peer review only

**ABSTRACT**

**Objective:** To explore the temporal changes of adolescents' views and experiences of participating in a gamified intervention to encourage physical activity behaviour, and associated processes of behaviour change.

**Design:** Longitudinal qualitative design. Focus groups were conducted with the same participants in each intervention school (n=3) at four time-points (baseline, end of each of two intervention phases and 1 year follow-up). The Framework method was used to thematically analyse the data.

**Setting:** Secondary schools (n=3), Belfast (Northern Ireland).

**Participants:** A sub-sample (n=19 at 4 time-points) of 12-14 year olds who participated in the StepSmart Challenge, a gamified intervention involving a pedometer competition and material rewards to encourage physical activity behaviour change.

**Results:** Three core themes were identified: 1) Competition; 2) Incentives and 3) Influence of friends. Participants indicated that a pedometer competition may help initiate physical activity, but suggested that there were a number of barriers such as participants finding it *"boring",* and feeling as though they had a remote chance of *"winning"*. ''Incentives" were viewed favourably, although there were participants who found not winning a prize *"annoying"*. Friends were a motivator to be more physically active, particularly for girls who felt encouraged to walk more when with a friend.

**Conclusions:** The intervention in general and specific gamified elements were generally viewed positively and deemed acceptable. Results suggest that gamification may have an important role to play in encouraging adolescents to engage in physical activity, and in creating interventions that are fun and enjoyable. The findings also suggest that gamified Behaviour Change Techniques align well with core concepts of Self-determination Theory, and that various game-elements may require tailoring for specific populations, for example, different genders.

**Trial Registration Number:** NCT02455986

**Strengths and limitations**

- A major strength of this study was the novel use of a longitudinal design, using the same participants in repeated focus groups. This enabled the investigation of how participants' views, experiences and PA behaviour evolved over time.

- In addition, having a number of researchers involved in the data collection and analyses reduces selectivity and researcher bias.
- However, only three focus groups participated in the study, and all were single sex schools.

For peer review only

## BACKGROUND

Physical activity (PA) levels in children and adolescents around the world are low. [1] As patterns of PA are established during this time and tend to track into adulthood, [2 3] this is a crucial period which can affect lifelong health and habits. To date, PA programmes for this population have shown limited effect, [4 5] stressing the need for innovative approaches to initiate and maintain PA behaviour. [6]

Programmes such as Pokémon GO illustrates the potential of gamified interventions (i.e. the application of elements of game playing, such as scoring points, competing against others, to change behaviour) for encouraging PA behaviour [7] and can provide useful insights into how to reach and engage the most inactive in PA behaviour. [8] Elements of gamification are incorporated into many commercial PA promotion apps, such as Pokémon GO, Fitocracy and 'Zombies, Run!', which include the collection of points for undertaking a targeted behaviour, completing challenges, or competing against others in virtual games. [9] Interventions that have applied gamification elements suggest it could be possible to make a routine activity such as travelling to school into a game that promotes active travel modes, and that is engaging and fun.[10 11]

Some key gamification strategies, including feedback on players' performance to allow them to set goals and monitor progression, competing with others, and use of incentives, are all evidence-based Behaviour Change Techniques (BCTs).[12] Further, research has demonstrated that other core aspects of gamified interventions such as opportunities for socialisation, self-evaluation, and rewarding positive behaviour are key to providing an enjoyable experience [13 14] and enjoyment has been identified as a significant predictor of PA behaviour.[15] However, gamification interventions have rarely been grounded in well-established theoretical frameworks and we know little about the views and experiences of participants.

The aim of this study was to explore the views and experiences of adolescents who participated in a gamified PA intervention based on Self-determination Theory, and the temporal changes of these views and experiences over the 1-year study period. The temporal analyses also enabled the investigation of potential behaviour change processes.

## METHODS

### Context

For peer review only

The StepSmart Challenge was a 24-week primarily school-based intervention utilising team, and individual competitions in five schools in Belfast, Northern Ireland. The school recruitment process is detailed elsewhere (Best et al; under review). An independent trial statistician randomly allocated the five schools to the intervention (three schools) or control (two schools) group. School characteristics are shown in Table 1; two were all-boys schools, two all-girls schools, and one was a co-educational school. All intervention schools were single sex (boys (n=1), girls (n=2)). Students (n=224) from Year 9 classes (aged 12-14 years) were invited to participate in the trial. The main results from the feasibility trial are published elsewhere (Best et al; under review). Briefly, the results demonstrated that the StepSmart Challenge was acceptable to young people for encouraging PA, and there was a trend in increasing light-intensity PA and improving mental wellbeing.

**The StepSmart Challenge**

The StepSmart Challenge was a gamified intervention designed according to Self-determination Theory (SDT),[16] using distinct intervention phases aiming to move participants along the motivation continuum from extrinsic motivation towards intrinsic motivation and encouraging PA behaviour change. The theory is grounded in three psychological needs – autonomy, competence, and relatedness. [16] Those intrinsically motivated engage in PA for the enjoyment and satisfaction it provides. [17] This form of motivation is associated with improved quality of life, increased PA behaviour, [18] and long-term behaviour change. [19] Self-motivation is undermined when individuals feel less control over the activity, and the environment, and if they do not feel a sense of connectedness or belonging to others engaging in the same activity. [20]

Table 2 details the various intervention components and links to BCTs. The intervention consisted of two phases. Phase One involved a multi-level (competition at the school, team and individual level) pedometer competition lasting for 8-weeks. Team selection was determined by the research team and took account of current PA levels and friendship networks measured at baseline; this was to ensure a mixed ability team (4-5 participants per team) with at least one friend in the team. The team competition comprised of social incentives such as publication of the results on the website, a trophy awarded to the leading team (i.e. the team with the highest number of total steps) in each school at competition end, and a prize to the winning school (£1000). During the individual competition in Phase One, incentives (approximate value of £10 – see Table 2 for details) were awarded weekly in

For peer review only - http://bmjopen.bmj.com/site/about/guidelines.xhtml

each school to two participants (participant who accumulated the most steps that week ('Walker of the Week'); and the participant who increased his/her step count the most from the previous week ('Most Improved')). Phase Two (14 weeks) focused on an individual level competition, in which the three participants that had accumulated the most steps in each school during this phase were awarded a 'goody bag' (approximate value of £30 for each participant and consisted of an assortment of those used in Phase One).

**Qualitative longitudinal research**

To elicit the temporal views and experiences of participants in the StepSmart Challenge a qualitative longitudinal research (QLR) design was used involving repeated semi-structured focus groups with the same participants over four data collection periods. This enabled qualitative charting of the perceived behaviour change process over a one-year period, and the opportunity to further understand potential mechanisms of behaviour change, and how perceptions and experiences of the intervention changed over time (pre, during and post-intervention). [21] Understanding why certain choices were made can produce more insightful and considered interpretation of behaviour change. [22] Such approaches are particularly valuable in providing a different perspective in assessing interventions or as part of process evaluations. [23]

**Focus group participants**

Baseline focus groups were conducted in each intervention school with a sub-sample of trial participants using a purposive sampling strategy whereby teachers identified potential participants with a range of PA levels from low to high to ensure data saturation. Those interested in taking part were given a study information sheet explaining the purpose of the focus groups, to read themselves and then give to their parent(s)/guardian(s). Parental (or guardian) opt-out consent and participant consent was sought from all participants. [24] Ethical approval was granted by the School of Medicine, Dentistry and Biomedical Science Research Ethics Committee (Queen's University, Belfast) (Ref: 15.09).

The focus groups were repeated during the study, following the same participants on their journey through the trial. This provided rich contextual data to explore the views and experiences of participants over time. Data were collected one-month pre-intervention (T0); at the end of the team competition (8 weeks) (T1); end of the individual competition (post-intervention) (24 weeks) (T2); and at 12-month follow-up (T3). Focus groups were conducted

For peer review only

on school premises and were audio recorded. The researchers verbally reaffirmed consent to participate at the beginning of each focus group. No other participants were present at the time of the focus groups.

Focus groups were semi-structured, based on topic guides (see Supplementary Material I) exploring core concepts at each time point. The topic guide was not piloted but developed iteratively reflecting on the data gathered from the focus groups from previous time points. Thus, emerging themes were explored across time points to chart changing views, experiences and PA behaviour. During all focus groups, the researcher summarised information at the end of each section and questioned understanding as a form of participant verification. [25]

Core concepts explored included:

1. General views and experiences of the intervention and intervention components;
2. Motivation to be active and to maintain being active long-term;
3. Extrinsic motivators including a) competition e.g. Does the competition motivate you to walk?; b) Material incentives e.g. Was the opportunity to win a prize something that motivated you?; c) Motivation for PA e.g. What motivates you to be active?;
4. SDT concepts including a) Autonomy e.g. What new ways have you found to be active?; b) Perceived competence e.g. How did it make you feel when you compared your steps to those of the class?; c) Relatedness e.g. Do you think friends are important in terms of how active you are?

Focus groups were conducted by RC (Male, PhD student), PB (Male, Post-doctoral Researcher) and RO'N (Female, Post-doctoral Researcher). PB and RO'N are experienced qualitative researchers and have facilitated focus groups with adolescents previously. RC had undergone a number of formal training courses in the facilitation of focus groups and thematic analysis methods. RC was accompanied to the focus groups by either RO'N or PB. No field notes were made during the focus groups. Saturation of the data was discussed between PB and RC. None of the researchers had any relationship with the participants.

**Data analysis**

Focus group recordings were transcribed verbatim and anonymised. Data were imported into NVivo (Version 10, QSR, Southport, UK) to manage and analyse the transcripts. Analysis

For peer review only

was undertaken using the Thematic Analysis Framework at the semantic level. [26] Initially researchers (RC and PB) familiarised themselves with the data. A sample coding frame was developed by the researchers independently, and refined iteratively with subsequent discussions. As a result, three coding frameworks were generated, one for each core theme. Illustrative quotes supporting emerging themes were highlighted and agreed by researchers. Transcripts were not returned to participants for comment, and they did not provided feedback on findings.

The dataset was systematically coded using an inductive approach and codes were generated to give a summary of elements of analytic interest. Once coded researchers identified potential themes from clusters of codes of similar meaning as well as patterns of responses across codes. Three central themes were identified at T0 and developed at subsequent time points. The coding frame was then discussed with ME and further refined. RC and ME then met multiple times to discuss and refine codes until a final coding frame was applied to all data. These themes consisted of (1) incentives; (2) competition; (3) influence of friends on PA. The temporal changes in the views and experiences of participants, and the influence of these components on the process of behaviour change were explored under each theme. Researchers (RC and ME) analysed the data together to further refine sub-themes to ensure assertions were accurately reflected.

**RESULTS**

Table 3 details the characteristics of participants in the focus groups, and demonstrates that the purposive sampling strategy was successful in recruiting participants of mixed gender, SES, from different teams, those who won prizes and those who didn't win, and PA levels. Table 3 displays a breakdown of the focus groups conducted. Twelve focus groups were conducted (mean duration 33 minutes; range 21–41 minutes). Focus group participants present at each time point varied due to participant absences (mean 6 participants; range 2–7 participants). No participants refused to participate or dropped out.

The following section details the themes and sub-themes identified from the thematic analysis.

**Theme 1: Competition**

For peer review only

Three sub-themes were identified including: a) usefulness of competition for PA behaviour change; b) self-competition for PA behaviour change; c) experiences of the team and individual competition.

*Sub-theme A: Usefulness of competition for PA behaviour change*

At T0, participants suggested the competition would motivate them to work harder and it was generally viewed as a motivating factor to become more physically active:

> *"that's what motivates me"* (School C, Male 5, T0).
> *"that's what pushes people on"* (School C, Male 6, T0).
> *"if I was actually in a competition I'd actually walk everywhere"* (School D, Female 6, T0).

Throughout the intervention, there appeared a sub-sample of participants in all schools who were motivated by more than just the desire to compete; the goal of winning was paramount:

> *"we like the winning but we don't like the losing"* (School E, Female 3, T0).
> *"to try and win like every week after that"* (School C, Male 6, T3).
> *"yeah, because you just wanted to win"* (School D, Female 6, T3).

For some participants, the competition was viewed as encouraging at the start but over time it became monotonous or 'boring':

> *"I think that's boring you know, who would want to know how many steps you're taking?"* (School D, Female 2, T2).
> *"at the start like, like you were quite motivated and then it just got more on and then you just forgot to wear it some days and it just got quite boring"* (School C, Male 4, T3).
> *"It was just like the same thing every day"* (School C, Male 4, T3).

Participants in School D (all girls) perceived their chances of winning the competition as remote, which suggests a lack of confidence in their capability in the competition.

For peer review only

> *"whenever you found out that you're actually losing there's just no point"* (School D, Female 2, T1).
> *"it's just sort of cause you knew you probably weren't going to win so you're just like there's really no point in wearing it [pedometer]"* (School D, Female 6, T2).
> *"well I just really gave up whenever X just won everything. I really did. I just stopped"* (School D, Female 3, T3).

*Sub-theme B: Perceptions of the usefulness of self-competition for PA behaviour change*

The potential use of the pedometer for self-directed goals, or 'self-competition' (competing against oneself) was considered promising at T0:

> *"if its showing you how many steps you're taking then you could challenge yourself to take more every day. So if you took 2000 steps one day you could try like try take more the next day. So it's like challenging yourself"* (School D, Female 1, T0).

Self-competition was shown to be a motivating factor throughout the intervention for most participants. One participant (School D) viewed 'getting better' and improving step counts as meaningful, reducing the negative effect of not winning prizes.

> *"trying to beat your day before target"* (School C, Male 5, T1).
> *"I loved to see like how many steps you were actually taking like when you're beating your scores as well like you check it one day and then the next day your gonna try and beat it"* (School E, Female 1, T2).
> *"No, it's alright because I was getting more each day so I was getting better; so it was alright"* (School D, Female 6, T3).

*Sub-theme C: Experiences of the team and individual competition*

The intervention incorporated two formats of a pedometer competition: a team-based competition (8 weeks duration), and an individual competition (14 weeks duration). During

For peer review only

the individual competition, participants competed against others from their school. At T0 the team-based competitions were seen to have the potential to better encourage PA:

> *"you can work together as a team"* (School D, Female 4, T0).
> *"if you were in like a group with more active people like you'd be sad that you're not as good as them but it would kind of push you to be as good as them"* (School E, Female 7, T0).

When asked to compare the individual with the team-based competition at T2, many participants from School C (all male) were more motivated by the team-based competition. This was due to the support provided by the team or peer pressure from not wanting to let *"your team down"*:

> *"I'd probably say it's easier with the support rather than individually"* (School C, Male 6, T2).
> *"you didn't want to let your team down"* (School C, Male 6, T2).

Reflecting on the intervention at T3, there were clear differences regarding experiences of the competition. School C (all boys) participants continued to feel positive about the team competition.

> *"the team's a lot better. Like its more… you are just like together"* (School C, Male 1, T3).
> *"The individual is quite boring"* (School C, Male 4, T3).

The girls' schools (School D and E) tended to favour the individual competition, as they did not have to rely on their team members, or for logistical reasons, such as the inability to meet up and 'organise stuff'.

> *"you don't have to depend on anyone else"* (School D, Female 3, T3).
> *"you know like sometimes you don't live close to your friends so you can't always organise stuff, which is a problem. So I think the individual one"* (School E; Female 7, T3).

For peer review only

One disadvantage of the team competition was the issue of free-riding team members (i.e. a member of a team that obtains benefits from membership but is not seen to contribute a fair share of the work needed to accrue the benefit).[27] In School D (all girls), free-riding was evident.

> *"well in the group you didn't really have to do anything cause the rest of them could do it but like by yourself like I don't know you just lose it altogether cause you don't walk"* (School D, Female 2, T2).
>
> *"we didn't really have to worry about it cause like the rest of them would've like walked anyway"* (School D, Female 2, T2).

**Theme 2: Incentives**

Two sub-themes were identified including a) type of incentive; and, b) perceptions of usefulness on incentives.

*Sub-theme A: Type of Incentive*

The provision of material incentives in this study was contingent on doing well in the competition, rather than being contingent on PA behaviour change. The types of incentives suggested and discussed by participants included recognition-based incentives (e.g. trophies) and material incentives (e.g. vouchers). Males tended to favour recognition based incentives, whereas material incentives with a higher monetary value were largely proposed by females. When asked what type of prizes they would like, male participants suggested:

> *"a medal or a trophy"* (School C, Male 5, T0).
>
> *"rugby ball"* (School C, Male 4, T0).

In contrast, females often suggested the use of material incentives.

> *"vouchers for clothes"* (School D, Female 6, T0).
>
> *"Topshop"* (School D, Female 1, T0).
>
> *"money"* (School E, Female 7, T0).

*Sub-theme B: Perceptions of usefulness of incentives*

For peer review only

At T1 and T2 many participants viewed incentives favourably. Participants suggested that the incentives were desirable and encouraged them more during the intervention.

> *"Every week cos you know it's like running out of time for like the prizes, just really want to get one"* (School C, Male 6, T1).
> *"that they weren't just like wee rubbish prizes they were really good ones"* (School E, Female 4, T2).

At T3, when reflecting on the intervention, participants still viewed the incentives as a motivating factor as they were 'good' prizes and provided acknowledgement for achievement:

> *"yeah, they look good. Like the prizes were really good"* (School C, Male 4, T3).
> *"yeah, I think [it] was good actually. Just to keep people motivated* (School E, Female 7, T3).
> *"like you know you are being acknowledged, like when you get prizes"* (School E, Female 3, T3).

Some participants did not win any prizes over the course of the intervention. When these participants discussed the instances when their peers won prizes there was a clear sense of disappointment, with a number stating that it was *"annoying":*

> *"kinda annoyed you when people like brought out their ten pound of cinema tickets and yeah it's like kinda annoying"* (School C, Male 3, T2).
> *"like it annoyed me that I didn't get one"* (School E, Female 6, T2).
> *"It just made me sad"* (School E; Female 2, T2).

**Theme 3: Influence of friends**

Two sub-themes emerged, including: a) the role of friends in general for encouraging PA behaviour, and b) the role of friends in team competition.

*Sub-theme A: Role of friends in general for encouraging PA behaviour*

For peer review only

At T0, participants suggested that PA was more enjoyable with friends, and the social support provided by friends encouraged participation in PA:

> *"it's about encouraging each other to do stuff"* (School C, Male 5, T0).
> *"if they want to go for a run you will want to go for a run with them"* (School C, Male 4, T0).
> *"Yeah, because you want to be doing it with them so you can enjoy yourself"* (School C, Male 6, T3)
> *"Good friends will help you yeah"* (School D, Female 6, T0).
> *"X only lives up the street so we go for runs most days after school"* (School E, Female 3, T0).

Participants in School E discussed the continued positive influence of friends on PA motivation at T1 and T2. Feeling 'scundered' (colloquialism for embarrassed) when walking alone is offset when provided the social support of friends:

> *"Make you feel like I'm going to be scundered [embarrassed] walking about alone but when you have your friend with you like you'd be more encouraged to do more walking if you're like walking with your friend"* (School E, Female 2, T2).
> *"hardly just like go a walk about yourself about the street like a big loner"* (School E, Female 4, T2).

*Sub-theme B: Role of friends in team competition*

Participants suggested that the influence of friends and a sense of connectedness was necessary within teams in order for them to work together and be competitive. Although, some participants in School C felt it would be *"good to have at least one friend or two"* (School C, Male 6, T3), they did not want to pick their own teams as they believed this might produce unbalanced teams with the more physically active individuals going into the same team. For these participants, the combination of friends within a team and homogeneity between teams was important:

> *"No cause then they could get really unfair"* (School C, Male 1, T2).

For peer review only

*"Cause all the active people could go in one team and then the inactive so it wouldn't work out"* (School C, Male 1, T2).

In contrast, participants in Schools D and E wanted the opportunity to choose team members; preferring to be in teams composed of their friends:

*"I wanted to choose my own team"* (School D, Female 4, T1)
*"no I think it should just be like your own group like friends like five of each of them"* (School E, Female 3, T1).
*"because like [if] you don't like people in your team you're just going to be like 'nah not even going to talk to you'"* (School E, Female 6, T2).

**DISCUSSION**

Participant's generally had positive experiences and views of a gamified PA intervention. Results suggested that the gamified design may have an important role to play in encouraging adolescents to engage in PA, and in creating interventions that are fun and enjoyable. The findings also suggested that core concepts of SDT are compatible with gamified BCTs, and that some game-elements may require tailoring for specific populations, for example, different genders.

In general, the use of a gamified pedometer competition was viewed favourably by participants. However, the goal of winning was very important for some and was key to sustaining their motivation to be active. This could be linked to the provision of material incentives which was contingent on 'winning' the competition. Over the course of the intervention, material incentives continued to motivate some participants, suggesting that intrinsic motivation and autonomy were not undermined. A possible mechanism could be that positive feedback provided by winning prizes and doing well in the competition, helped develop an individual's intrinsic motivation by improving feelings of competence. [28] The positive effect of material incentives for health behaviour change with children and adolescents has also been shown in previous studies. [29-32] and Corepal et al (under review).

The long-term effect is less clear with some studies showing that positive effects dissipates over time. [33] This could be due to habitation to the extrinsic motivators being offered [34] or a

For peer review only

'crowding out effect' of intrinsic motivation [35] once extrinsic incentives are removed. However, to date this hypothesis has not been tested or supported in 'real world' interventions. [28 36-38]

Other participants suggested feeling de-motivated from the outset as they felt they had no chance of winning. Some became less enthusiastic about the competition if other participants consistently had a greater number of steps, and were disappointed at not winning a prize. Previous work has suggested that competition can affect participants' self-evaluation of their competence to perform the task. [39 40] If a participant loses, and their loss is attributed to low ability, this can negatively impact behaviour. [41] Therefore, as a preventative measure, participants may choose not to compete, or not engage in the competition with maximum effort. [42] This helps to provide some explanation for the loss, other than low ability, and thus preserving the participant's self-esteem and self-efficacy.

Some participants indicated that they became gradually less interested because of the repetitive nature of the pedometer competition. These findings are supported by a large body of literature which suggests that extrinsic motivators can have a short-term positive effect on motivation which is not maintained. [33 36 37 43] Extrinsic motivators such as competition and material incentives could be used to initially stimulate the interest of participants, especially those with lower levels of PA. [44] However, a key learning point would be to transition to more intrinsically motivating forms of PA and thus the incorporation of BCTs that focus on these behaviours would be useful. [45 46]

The competition had various levels: rewards could be offered to the highest achieving team, the highest achieving individual, or to anyone on the basis of achieving some personal goals (self incentive). The findings showed distinct perceptions regarding the value of each. For example, males tended to prefer the team competition and suggested they would try harder to contribute to the team, and found the team environment supportive and enjoyable. Maculada [47] suggested that males find team affiliation important, and a way to be accepted by peers and to feel a sense of belonging with the group. Team-based PA interventions have been shown to be effective, [10 11] and may be less harmful than individual competitions. [48] Conversely, females favoured individual competition; how well a participant did in the competition was not dependent on the effort of others, mitigating to the problem of free-riding. [49] One solution may be to distribute incentives equitably (ie proportionate to effort

For peer review only - http://bmjopen.bmj.com/site/about/guidelines.xhtml

and contribution) to team members rather than distributing them equally, [50] thereby reducing free-riding and increasing effort. [51 52]

Self-competition [53] was seen as a prominent positive influence of PA. Participants often used the pedometers for feedback, to self-monitor, and set personal step goals. Creating achievable personal goals may also play a part in mitigating the potential negative effects of extrinsic motivators by emphasising competence (by meeting goals and receiving positive feedback), autonomy (as participants are free to choose which activities they pursue to increase step counts), and maintaining self-efficacy. Self-competition with the use of intrinsic goals was enjoyed by all participants in the focus groups, regardless of success in the overall competition. Self-competition allowed participants to be autonomous, and to create achievable challenges such as walking more steps than during the previous day. Therefore, self-competition could be a way to develop autonomous identified or integrated regulation, which has been shown to have benefits for PA motivation. [54 55] Autonomy-supportive elements such as self-competition could consequently stimulate the development of habit formation. [56]

The significance of friends for influencing PA behaviour has also been frequently cited in the literature. [57-59] The participants' feelings on team composition and the influence of friends reinforce the physiological need for relatedness, a core construct of SDT. Participants from all schools felt that a sense of connectedness to the group was important for an effective team competition. Other research shows that adolescents value opportunities for social interaction, [39] and so team membership could have a positive effect on PA motivation. Participants stated that friends provide support, encouragement, and help with the enjoyment of PA.

**Strengths and limitations**

A major strength of this study was the novel use of a longitudinal design, [21 60] using the same participants in repeated focus groups at baseline, post-intervention and 1-year follow-up. This enabled the investigation of how participants' views, experiences and PA behaviour evolved over time. The findings are robust as assumptions, views and experiences can be tested and re-tested in subsequent sessions, and researchers build relationships with participants due to the repeated exposure which can encourage disclosure. In addition,

For peer review only

having a number of researchers involved in the data collection and analyses reduces selectivity and researcher bias.

Focus groups were chosen as an appropriate approach as they provide opportunity for the group to discuss issues amongst themselves and reach consensus, gathering multiple viewpoints and representing 'everyday' conversation. However, they have been criticised for lacking depth, particularly when conducted with young people as they tend not to elaborate on points. It may also have been useful to combine this approach with 1:1 interviews to reduce peer pressure and ensure coherency of responses at different schools. Finally, only three focus groups participated in the study, and all were single sex schools. Consequently the purported gender differences may be an artefact of differences in socio-economic status as well as or in addition to gender differences.

**Conclusions**

Preferences for gamified elements including team or individual competitions, and the influence of friends on PA behaviour were highlighted. The use of a longitudinal qualitative design enabled exploration of temporal changes in participants' views and experiences, and exploration of potential mechanisms of behaviour change. This study suggests that the three core constructs for self-motivation in SDT could be important factors for motivating PA in adolescents via competition and the use of material rewards delivered through gamification. This supports previous research which proposes benefits in providing opportunities for autonomy, perceived competence, and relatedness. [61]

**Acknowledgments:** The authors wish to thank the pupils and teachers who were involved in the development of this intervention, and those who participated in the study.

**Competing Interests:** The authors declare that they have no competing interests.

**Data Sharing Statement:** No additional data are available.

**Funding:** The authors wish to acknowledge funding from a HSC R&D (NI) Enabling Research Award. RH is supported by a NIHR Career Development Fellowship and acknowledges funding support from the HSC Research and Development Division. The work was undertaken under the auspices of the UKCRC Centre of Excellence for Public Health Research Northern Ireland and Centre for Diet and Activity Research (CEDAR), UKCRC Public Health

For peer review only

Research Centres of Excellence which are funded by the British Heart Foundation, Cancer Research UK, Economic and Social Research Council, Medical Research Council, the National Institute for Health Research, and the Wellcome Trust. The funders had no role in study design, data collection, data analysis, data interpretation, or writing of the report.

For peer review only

**References**

1. Tremblay MS, Barnes JD, González SA, et al. Global Matrix 2.0: Report Card Grades on the Physical Activity of Children and Youth Comparing 38 Countries. *J Phys Act Health* 2016;13(11 Suppl 2):S343-S66. doi: 10.1123/jpah.2016-0594

2. Telama R. Tracking of physical activity from childhood to adulthood: a review. *Obes Facts* 2009;2(3):187-95. doi: 10.1159/000222244 [published Online First: 2009/06/12]

3. Telama R, Yang X, Leskinen E, et al. Tracking of physical activity from early childhood through youth into adulthood. *Medicine and science in sports and exercise* 2014;46(5):955-62. doi: 10.1249/MSS.0000000000000181

4. Metcalf B, Henley W, Wilkin T. Effectiveness of intervention on physical activity of children: systematic review and meta-analysis of controlled trials with objectively measured outcomes (EarlyBird 54). *BMJ (Clinical research ed)* 2012;345:e5888.

5. Soares NM, Leão AS, Santos JR, et al. Systematic review shows only few reliable studies of physical activity intervention in adolescents. *ScientificWorldJournal* 2014;2014:206478. doi: 10.1155/2014/206478 [published Online First: 2014/07/20]

6. Gillis L, Tomkinson G, Olds T, et al. Research priorities for child and adolescent physical activity and sedentary behaviours: an international perspective using a twin-panel Delphi procedure. *The international journal of behavioral nutrition and physical activity* 2013;10:112. doi: 10.1186/1479-5868-10-112 [published Online First: 2013/10/24]

7. McCartney M. Margaret McCartney: Game on for Pokémon Go. *BMJ (Clinical research ed)* 2016;354:i4306. [published Online First: 2016/08/09]

8. Wong FY. Erratum to: Influence of Pokémon Go on physical activity levels of university players: a cross-sectional study. *Int J Health Geogr* 2017;16(1):17. doi: 10.1186/s12942-017-0089-5 [published Online First: 2017/04/26]

9. Lister C, West JH, Cannon B, et al. Just a fad? Gamification in health and fitness apps. *JMIR Serious Games* 2014;2(2):e9. doi: 10.2196/games.3413 [published Online First: 2014/08/04]

10. Hunter RF, de Silva D, Reynolds V, et al. International inter-school competition to encourage children to walk to school: a mixed methods feasibility study. *BMC research notes* 2015;8:19. doi: 10.1186/s13104-014-0959-x [published Online First: 2015/01/28]

11. Coombes E, Jones A. Gamification of active travel to school: A pilot evaluation of the Beat the Street physical activity intervention. *Health Place* 2016;39:62-9. doi: 10.1016/j.healthplace.2016.03.001 [published Online First: 2016/03/11]

12. Michie S, Richardson M, Johnston M, et al. The behavior change technique taxonomy (v1) of 93 hierarchically clustered techniques: building an international consensus for the reporting of behavior change interventions. *Ann Behav Med* 2013;46(1):81-95. doi: 10.1007/s12160-013-9486-6 [published Online First: 2013/03/21]

For peer review only

13. Sardi L, Idri A, Fernandez-Aleman JL. A systematic review of gamification in e-Health. *Journal of biomedical informatics* 2017;71:31-48. doi: 10.1016/j.jbi.2017.05.011 [published Online First: 2017/05/26]

14. Cugelman B. Gamification: what it is and why it matters to digital health behavior change developers. *JMIR Serious Games* 2013;1(1):e3. doi: 10.2196/games.3139 [published Online First: 2013/12/12]

15. Best P, Tully MA, Corepal R, et al. Time to 're-think' physical activity promotion for young people? Results from a repeated cross-sectional study. *BMC Public Health* 2017;17(1):208. doi: 10.1186/s12889-017-4136-8 [published Online First: 2017/02/17]

16. Ryan RM, Deci EL. Self-determination theory and the facilitation of intrinsic motivation, social development, and well-being. *The American psychologist* 2000;55(1):68-78.

17. Ryan RM, Williams GC, Patrick H, et al. Self-determination theory and physical activity: The dynamics of motivation in development and wellness. *Hellenic Journal of Psychology* 2009;6(2):107-24.

18. Ryan RM, Patrick H, Deci EL, et al. Facilitating health behaviour change and its maintenance: Interventions based on self-determination theory. *European Health Psychologist* 2008;10(1):2-5.

19. Silva MN, Marques MM, Teixeira PJ. Testing theory in practice: The example of self-determination theory-based interventions. *Eur Health Psychol* 2014;16(5):171-80.

20. Sebire SJ, Kesten JM, Edwards MJ, et al. Using self-determination theory to promote adolescent girls' physical activity: Exploring the theoretical fidelity of the Bristol Girls Dance Project. *Psychol Sport Exerc* 2016;24:100-10. doi: 10.1016/j.psychsport.2016.01.009

21. Saldana J. Longitudinal Qualitative Research: Analysing change through time. California: AltaMira Press 2003.

22. Corden A, Millar J. Qualitative longitudinal research for social policy - Introduction to themed section. *Social Policy and Society* 2007;6(4):529-32.

23. Calman L, Brunton L, Molassiotis A. Developing longitudinal qualitative designs: lessons learned and recommendations for health services research. *BMC Med Res Methodol* 2013;13:14. doi: 10.1186/1471-2288-13-14 [published Online First: 2013/02/06]

24. Jago R, Bailey R. Ethics and paediatric exercise science: issues and making a submission to a local ethics and research committee. *J Sports Sci* 2001;19(7):527-35. doi: 10.1080/026404101750238980

25. Harper M, Cole P. Member checking: can benefits be gained similar to group therapy? *The Qualitative Report* 2012;17(2):510-17.

26. Braun V, Clarke V. Using thematic analysis in psychology. *Qualitative Research in Psychology* 2006;3(2):77-101.

For peer review only

27. Albanese RvF, David D. Rational Behavior in Groups: The Free-Riding Tendency. *Academy of Management Review* 1985;10(2):244-55. doi: 10.5465/AMR.1985.4278118

28. Barte JC, Wendel-Vos GC. A Systematic Review of Financial Incentives for Physical Activity: The Effects on Physical Activity and Related Outcomes. *Behav Med* 2015:1-12. doi: 10.1080/08964289.2015.1074880

29. Suchert V, Isensee B, Sargent J, et al. Prospective effects of pedometer use and class competitions on physical activity in youth: A cluster-randomized controlled trial. *Prev Med* 2015;81:399-404. doi: 10.1016/j.ypmed.2015.10.002

30. Hardman CA, Horne PJ, Fergus Lowe C. Effects of rewards, peer-modelling and pedometer targets on children's physical activity: a school-based intervention study. *Psychology & health* 2011;26(1):3-21. doi: 10.1080/08870440903318119

31. Horne PJ, Hardman CA, Lowe CF, et al. Increasing children's physical activity: a peer modelling, rewards and pedometer-based intervention. *Eur J Clin Nutr* 2009;63(2):191-8. doi: 10.1038/sj.ejcn.1602915

32. Finkelstein EA, Tan YT, Malhotra R, et al. A cluster randomized controlled trial of an incentive-based outdoor physical activity program. *J Pediatr* 2013;163(1):167-72.e1. doi: 10.1016/j.jpeds.2013.01.009

33. Cahill K, Hartmann-Boyce J, Perera R. Incentives for smoking cessation. *Cochrane Database Syst Rev* 2015(5):CD004307. doi: 10.1002/14651858.CD004307.pub5

34. Burns RJ, Donovan AS, Ackermann RT, et al. A theoretically grounded systematic review of material incentives for weight loss: implications for interventions. *Ann Behav Med* 2012;44(3):375-88. doi: 10.1007/s12160-012-9403-4

35. Frey BS, Jegen R. Motivation Crowding Theory. *Journal of Economic Surveys* 2001;15(5):589-611. doi: 10.1111/1467-6419.00150

36. Mantzari E, Vogt F, Shemilt I, et al. Personal financial incentives for changing habitual health-related behaviors: A systematic review and meta-analysis. *Prev Med* 2015;75:75-85. doi: 10.1016/j.ypmed.2015.03.001 [published Online First: 2015/04/07]

37. Mitchell MS, Goodman JM, Alter DA, et al. Financial incentives for exercise adherence in adults: systematic review and meta-analysis. *Am J Prev Med* 2013;45(5):658-67. doi: 10.1016/j.amepre.2013.06.017

38. Strohacker K, Galarraga O, Williams DM. The impact of incentives on exercise behavior: a systematic review of randomized controlled trials. *Ann Behav Med* 2014;48(1):92-9. doi: 10.1007/s12160-013-9577-4

39. Knowles AM, Niven A, Fawkner S. A qualitative examination of factors related to the decrease in physical activity behavior in adolescent girls during the transition from primary to secondary school. *J Phys Act Health* 2011;8(8):1084-91.

40. Ames C. Achievement Attributions and Self-Instructions Under Competitive and Individualistic Goal Structures. *Journal of Educational Psychology* 1984;76(3):478-87.

For peer review only

41. Lam SF, Yim PS, Law JS, et al. The effects of competition on achievement motivation in Chinese classrooms. *Br J Educ Psychol* 2004;74(Pt 2):281-96. doi: 10.1348/000709904773839888

42. Grant D, Green W. Grades as incentives. *Empirical Economics* 2013;44(3):1563-92.

43. Giles EL, Robalino S, McColl E, et al. The effectiveness of financial incentives for health behaviour change: systematic review and meta-analysis. *PLoS One* 2014;9(3):e90347. doi: 10.1371/journal.pone.0090347 [published Online First: 2014/03/13]

44. Hunter RF, Tully MA, Davis M, et al. Physical activity loyalty cards for behavior change: a quasi-experimental study. *Am J Prev Med* 2013;45(1):56-63. doi: 10.1016/j.amepre.2013.02.022

45. Seghers J, Vissers N, Rutten C, et al. Intrinsic goals for leisure-time physical activity predict children's daily step counts through autonomous motivation. *Psychology of Sport and Exercise* 2014;15(3):247-54. doi: 10.1016/j.psychsport.2014.01.003

46. Sebire SJ, Jago R, Fox KR, et al. Testing a self-determination theory model of children's physical activity motivation: a cross-sectional study. *The international journal of behavioral nutrition and physical activity* 2013;10:111. doi: 10.1186/1479-5868-10-111 [published Online First: 2013/09/26]

47. Maculada ST. Gender Differences in Task and Ego Goal Orientation and Motivation in Sport Participation. *Philippine Journal of Psychology* 2007;40(1)

48. Effects of competition in education: A case study in an e-learning environment. Proceedings of the IADIS International Conference E-learning; 2010.

49. Maiden B, Perry B. Dealing with free‐riders in assessed group work: results from a study at a UK university. *Assessment & Evaluation in Higher Education* 2011;36(4):451-64.

50. Garbers Y, Konradt U. The effect of financial incentives on performance: A quantitative review of individual and team‐based financial incentives. *Journal of Occupational and Organizational Psychology* 2013;87(1):102-37. doi: 10.1111/joop.12039

51. Gunnthorsdottir A, Rapoport A. Embedding social dilemmas in intergroup competition reduces free-riding. *Organizational Behavior and Human Decision Processes* 2006;101(2):184-99.

52. Kugler T, Rapoport A, Pazy A. Public good provision in inter‐team conflicts: Effects of asymmetry and profit‐sharing rule. *Journal of Behavioral Decision Making* 2010;23(4):421-38.

53. Howe LA. On competing against oneself, Or 'I need to get a different voice in my head'. *Sport, ethics and Philosophy* 2008;2(3):353-66. doi: Sports Ethics and Philosophy, Vol. 2, No. 3, December 2008, pp. 353–366

54. Van den Berghe L, Vansteenkiste M, Cardon G, et al. Research on self-determination in physical education: key findings and proposals for future research. *Physical*

For peer review only

*Education and Sport Pedagogy* 2014;19(1):91-121. doi: info:doi/10.1080/17408989.2012.732563

55. Teixeira PJ, Carraça EV, Markland D, et al. Exercise, physical activity, and self-determination theory: a systematic review. *The international journal of behavioral nutrition and physical activity* 2012;9:78. doi: 10.1186/1479-5868-9-78 [published Online First: 2012/06/22]

56. Gardner B, Lally P. Does intrinsic motivation strengthen physical activity habit? Modeling relationships between self-determination, past behaviour, and habit strength. *J Behav Med* 2013;36(5):488-97. doi: 10.1007/s10865-012-9442-0 [published Online First: 2012/07/04]

57. Fitzgerald A, Fitzgerald N, Aherne C. Do peers matter? A review of peer and/or friends' influence on physical activity among American adolescents. *J Adolesc* 2012;35(4):941-58. doi: 10.1016/j.adolescence.2012.01.002

58. Maturo CC, Cunningham SA. Influence of friends on children's physical activity: a review. *Am J Public Health* 2013;103(7):e23-38. doi: 10.2105/ajph.2013.301366 [published Online First: 2013/05/18]

59. Sawka KJ, McCormack GR, Nettel-Aguirre A, et al. Friendship networks and physical activity and sedentary behavior among youth: a systematized review. *The international journal of behavioral nutrition and physical activity* 2013;10:130. doi: 10.1186/1479-5868-10-130

60. Lewis J. Analysing Qualitative Longitudinal Research in Evaluations. *Social Policy and Society* 2007;6(4):545-56. doi: 10.1017/S1474746407003880 [published Online First: 10/01]

61. MacPhail A, Gorely T, Kirk D, et al. Children's experiences of fun and enjoyment during a season of sport education. *Research Quarterly for Exercise and Sport* 2008;79(3):344-55.

For peer review only

**Table 1: Characteristics of schools included in the StepSmart Challenge feasibility study**

| | **Intervention or Control Group** | **Single Sex or Co-educational** | **Free school meal entitlement (%)** |
|---|---|---|---|
| **School A** | Control | All Male | 63.7% |
| **School B** | Control | Co-Educational | 7.2% |
| **School C** | Intervention | All Male | 8.0% |
| **School D** | Intervention | All Female | 56.5% |
| **School E** | Intervention | All Female | 54.6% |

For peer review only

**Table 2: Intervention Components and Behaviour Change Techniques (BCTs)**

| **Component** | **Activity/Task** | **Behavioural Change Technique** |
|---|---|---|
| Competition | Competition was designed to take place across three levels during Phase one (April – June 2015).<br><br>**1.** School level - £1000 prize for winning school<br>**2.** Team level – trophy for the winning team in each school<br>**3.** Individual level – weekly prizes for highest steps and most improved within each school<br><br>During Phase two (July – Sept 2015) there were individual prizes for the top three participants in each school achieving the highest average number of steps across the 14-week period. This two-phased tapered approach was designed to encourage med-long term PA behaviour change (i.e. extrinsic to intrinsically motivated PA behaviour) | -Set graded tasks<br>-Provide rewards contingent on successful behaviour<br>-Provide feedback on performance |
| Material Rewards/Prizes | Material rewards included coloured stickers, selfie sticks, completion certificates, cinema tickets and £10 sports vouchers. Individual prizes were awarded on a weekly basis under two categories, 'outstanding performance' and 'most improved'. | -Prompt rewards contingent on effort or progress towards behaviour |
| Teams | A team based competition was developed alongside the main school competition to encourage peer support. Ten teams were created within each school (4-5 participants per team). Team captains were selected based on baseline PA data to ensure balance between teams and peer nominations to identify those 'most looked up to'. The highest placed team within each school at the end of Phase One was awarded with a trophy. | -Plan social support/ social change<br>-Facilitate social comparison<br>-Prompt identification as role model/ position advocate |
| Pedometers | Participants were given a Fitbit Zip pedometer and asked to wear throughout every day of the intervention (Phase One | -Goal setting (outcome)<br>-Prompt self-monitoring of behavioural outcome |

For peer review only

| | | |
|---|---|---|
| | and Two). Pedometers provided participants with feedback on daily steps and were uploaded to the study website via the Fitbit App or using a wireless dongle located at designated areas within schools. | -Provide feedback on performance |
| Website | Pedometer data was uploaded to the StepSmart Challenge website and participants could review their daily/weekly scores and view the competition leader board. The website included the provision of motivational messages, weekly challenges and links to other PA resources | -Goal setting (outcome)<br>-Prompt self-monitoring of behavioural outcome<br>-Provide feedback on performance |
| Workbook | A short workbook was given to participants at the start of the intervention. This included 'fun-facts', tips and challenges to promote PA behaviour as well as a section for the participant to record weekly step target (individual and team). | -Provide information on consequences of behaviour in general<br>-Goal setting (outcome)<br>-Prompting generalization of a target behaviour |

For peer review only

**Table 3: Characteristics of focus group participants**

| | | | Average steps per day (measured using accelerometers) | | |
|---|---|---|---|---|---|
| **Participant** | | **Team** | **Baseline** | **Post-intervention** | **12 Month Follow Up** |
| 1 | **School C** | C10 | 9,949 | 8,576 | No valid data |
| 2 | **School C** | C6 | No valid data | No valid data | No valid data |
| 3 | **School C** | C6 | 8,815 | 13,127 | No valid data |
| 4 | **School C** | C7 | 9,325 | 4,099 | 4,099 |
| 5 Winner of 'Most Improved' | **School C** | C1 | 9,264 | 6,687 | 14,246 |
| 6 Winner of 'Walker of the Week' Winner of Summer Competition | **School C** | C5 | 13,326 | 9,563 | 8,039 |
| 1 Winner of 'Walker of the Week' Winner of Summer Competition | **School D** | D2 | 10,940 | 10,684 | 11,784 |
| 2 | **School D** | D9 | 2,787 | No valid data | No valid data |
| 3 Winner of 'Most Improved' | **School D** | D6 | 9,737 | 7,160 | 7,160 |
| 4 | **School D** | D5 | 6,555 | No valid data | 4,088 |
| 5 | **School D** | D5 | 2,782 | No valid data | 5,426 |
| 6 Winner of 'Most Improved' | **School D** | D7 | 9,253 | No valid data | No valid data |
| 1 | **School E** | E7 | 6,495 | 13,080 | 6,129 |
| 2 | **School E** | E7 | 7,330 | No valid data | 9,440 |
| 3 | **School E** | E2 | 6,583 | No valid data | No valid data |
| 4 | **School E** | E9 | 5,915 | No valid data | No valid data |
| 5 Winner of 'Walker of the Week' Winner of Summer Competition | **School E** | E6 | 14,153 | 13,998 | 8,179 |
| 6 Winner of 'Most Improved' | **School E** | E3 | 14,113 | No valid data | 9,988 |
| 7 Winner of 'Walker of the Week' Winner of Summer Competition | **School E** | E3 | 11,330 | No valid data | 5,909 |

* No valid data = Unreturned accelerometer or no valid three-day measurement of data

For peer review only

**Table 4: Overview of the number of participants in (and duration of) each focus group at each time point**

| | **Time points of each focus group** | | | |
|---|---|---|---|---|
| **Intervention schools** | *Baseline (T0)* | *8 weeks (T1)* | *24 weeks (T2)* | *52 weeks (T3)* |
| **School C (All Boys)** | 6 (35 mins) | 6 (21 mins) | 5 (38 mins) | 5 (35 mins) |
| **School D (All Girls)** | 6 (34 mins) | 5 (37 mins) | 6 (40 mins) | 2 (31 mins) |
| **School E (All Girls)** | 7 (36 mins) | 7 (41 mins) | 6 (24 mins) | 7 (24 mins) |

For peer review only

**Supplementary Material I: Topic Guide**

| Topic Guide | | | |
|---|---|---|---|
| **Pre-intervention** | **End of phase one (External regulation; Introjected regulation)** | **End of phase two (Introjected regulation; Identified regulation)** | **12 months post-baseline follow up (Integrated motivation)** |
| **Explore the barriers and facilitators to PA**<br><br>What stops you from being physically active? (Relational support)<br><br>Role of parents/friends in your PA? (Autonomy support; Relational support)<br><br>Any ideas to increase PA? (Autonomy support)<br><br>Opportunities for PA within school/community? (Autonomy support; Relational support) | **Explore the experiences of StepSmart**<br><br>How easy is it to find ways in which to be more active? (Autonomy support)<br><br>Did completing the StepSmart Challenge with friends make it easier? Or more pressure? (Autonomy support; Relational support)<br><br>Do you enjoy telling people about your success in terms of PA (e.g. showing others your steps)? (Perceived competence; Relatedness) | **Explore the how participants felt about the different competition elements**<br><br>How did the team competition compare to the individual competition? (Relational support)<br><br>Some of the class were more/less active than you. How did you feel when you compared your steps to the class? (Perceived competence)<br><br>Did team members encourage you to be active or not? (Autonomy support)<br><br>How did you feel if you didn't contribute to the team/school's step count? (Perceived competence) | **Explore if there has been a change in PA (formation of habit)**<br><br>One year on: how active are you now (compared to before you took part)?<br><br>Did you make changes to your PA? Why/why not? *(changes to routine ... active travel, walking with friends etc.)*<br><br>If you did make any changes, which are you still doing? What strategies helped? (Autonomy support) |
| **Explore the acceptability of the intervention components**<br><br>Have you ever heard of…or used a pedometer?<br>What is it you (dis)like about them?<br><br>Feelings on writing down your own daily steps goals in a workbook. | **Explore the attitude towards PA**<br><br>Do you enjoy PA?<br><br>What are your main reasons for being active? (i.e. role of the prizes and other elements?)<br><br>Do you enjoy finding new | **Explore how the StepSmart Challenge instigated any changes in participants PA**<br><br>How do you feel about PA since the StepSmart Challenge?<br><br>What have you enjoyed?<br><br>Has the StepSmart Challenge increased your PA? If not, what would motivate you | **Explore how participants felt about the StepSmart Challenge**<br><br>Why did you choose to participate?<br><br>Good/not so good aspects of the competition?<br><br>Did the competition motivate – no/at beginning/all throughout? |

For peer review only



For peer review only - http://bmjopen.bmj.com/site/about/guidelines.xhtml

| | | | |
|---|---|---|---|
| (Perceived competence; Autonomy support)<br><br>What features of a website would you find appealing?<br><br>How do you feel about entering a school-based competition?<br><br>Any advantages/disadvantages to team vs individual competitions<br><br>What prizes would motivate young people to take part? | ways of becoming more active?<br>(Autonomy support)<br><br>Do you think your motivation towards PA has changed? | to be more active?<br><br>Since the StepSmart Challenge, what new opportunities have you found to be active?<br>(Autonomy support)<br><br>Anything outside your control stopping you from being active? (e.g. park proximity, family, other)<br>(Autonomy support; Relational support) | |
| | **To explore the consequences of PA**<br><br>Do you find yourself having more energy?<br><br>Do you feel more confident in being able to achieve things?<br>(Perceived competence)<br><br>How do you feel after you've completed some PA?<br><br>Do you think you are more active now than when you started? Why? | **Explore how participants felt about the use of the pedometer**<br><br>How often did you use the pedometer? What did you like/dislike about it? (e.g. Feedback)<br>(Perceived competence; Autonomy support)<br><br>How many of you are still using the pedometer? Why? | **Explore how participants felt about the team/individual competition**<br><br>Class vs school, was one enjoyed more?<br>(Autonomy support; Relational support)<br><br>Influence of friends on being active?<br>(Autonomy support; Relational support)<br><br>Opportunity to make new friends?<br>(Relational support)<br><br>Feelings about not contributing to the team/school step count?<br>(Perceived competence)<br><br>More motivated if friends wanted to do well in the competition?<br>(Relational support) |



For peer review only

For peer review only - http://bmjopen.bmj.com/site/about/guidelines.xhtml

| | | | |
|---|---|---|---|
| | | **Explore how participants felt about the rewards/prizes**<br><br>What did you think of the prizes?<br><br>Tell me how well they worked to motivate you?<br><br>How did you feel if you didn't win a prize? (Perceived competence)<br><br>If no prizes, but still a competition, would you still be as active? Why? | **Explore how participants felt about the rewards/prizes**<br><br>Type of prizes preferred, including ttrophy or a certificate?<br><br>Not competing against others, but beating your own goal? (Autonomy support; Perceived competence) |
| | | **Explore how the website and Facebook group were used**<br><br>Things you liked/didn't like about the website?<br><br>What did you think of Facebook group? (Autonomy support; Relational support)<br><br>Which way would you prefer to get your information from? (Autonomy support) | **Mood/motivation did these change at different stages of the intervention?**<br><br>Feelings if didn't make as many steps as others? Did that affect your mood? (Perceived competence; Relational support)<br><br>If you did/didn't do well - did that motivate/demotivate you? (Perceived competence; Relational support)<br><br>If felt like don't have a good chance to win would that make you stop trying? (Perceived competence; Relational support) |

Red text illustrates how items in the topic guide are linked the concepts of self-determination theory



For peer review only - http://bmjopen.bmj.com/site/about/guidelines.xhtml

# Consolidated criteria for reporting qualitative studies (COREQ): 32-item checklist

Developed from:
Tong A, Sainsbury P, Craig J. Consolidated criteria for reporting qualitative research (COREQ): a 32-item checklist for interviews and focus groups. *International Journal for Quality in Health Care*. 2007. Volume 19, Number 6: pp. 349 – 357

| No. Item | Guide questions/description |
|---|---|
| **Domain 1: Research team and reflexivity** | |
| *Personal Characteristics* | |
| 1. Inter viewer/facilitator | Which author/s conducted the interview or focus group?<br>**PB, RO'N, RC** |
| 2. Credentials | What were the researcher's credentials? E.g. PhD, MD<br>**PhD (PB), PhD (RO'N), MPH (RC)** |
| 3. Occupation | What was their occupation at the time of the study?<br>**PB: Research Fellow, UKCRC Centre of Excellence for Public Health/Centre for Public Health**<br>**RO: Research Fellow, UKCRC Centre of Excellence for Public Health/Centre for Public Health**<br>**RC: PhD Student** |
| 4. Gender | Was the researcher male or female?<br>**Male (PB), Female (RO'N), Male (RC)** |
| 5. Experience and training | What experience or training did the researcher have?<br>**PB: Qualified social worker, training in thematic analysis, Computer Assisted Qualitative Data Analysis (CAQDAS) packages**<br>**RO'N: Experience in the design, conduct and analysis of a photo elicitation and focus group based qualitative research. Training in focus group facilitation and the NVivo**<br>**RC: MPH with a focus on health services research comprising training on qualitative research methods. Formal training in NVivo during PhD** |
| *Relationship with participants* | None |
| 6. Relationship established | Was a relationship established prior to study commencement?<br>**No** |
| 7. Participant knowledge of the interviewer | What did the participants know about the researcher? e.g. personal goals, reasons for doing the research<br>**PB recruited interviewees to the study** |
| 8. Interviewer characteristics | What characteristics were reported about the inter viewer/facilitator? e.g. Bias, assumptions, reasons and interests in the research topic<br>**None** |
| **Domain 2: study design** | |

| | |
|---|---|
| *Theoretical framework* | |
| 9. Methodological orientation and Theory | What methodological orientation was stated to underpin the study? e.g. grounded theory, discourse analysis, ethnography, phenomenology, content analysis<br>**Underpinned by Self-determination theory. Framework Method was used for qualitative data analysis** |
| *Participant selection* | |
| 10. Sampling | How were participants selected? e.g. purposive, convenience, consecutive, snowball<br>**Purposive sampling used for participant focus groups** |
| 11. Method of approach | How were participants approached? e.g. face-to-face, telephone, mail, email<br>**Face-to-face** |
| 12. Sample size | How many participants were in the study?<br>19 participants<br>**School C (n = 6); School D (n = 6); School E (n = 7)** |
| 13. Non-participation | How many people refused to participate or dropped out? Reasons?<br>**None** |
| *Setting* | |
| 14. Setting of data collection | Where was the data collected? e.g. home, clinic, workplace<br>**Data was collected in each intervention school (n = 3)** |
| 15. Presence of non-participants | Was anyone else present besides the participants and researchers?<br>**No** |
| 16. Description of sample | What are the important characteristics of the sample? e.g. demographic data, date<br>**Year 9 classes from 3 schools over 2015-2016**<br>**School C: All male aged between 12 to 14 years**<br>**School D: All female aged between 12 to 14 years**<br>**School E: All female aged between 12 to 14 years** |
| *Data collection* | |
| 17. Interview guide | Were questions, prompts, guides provided by the authors?<br>**Yes**<br>Was it pilot tested?<br>**No** |
| 18. Repeat interviews | Were repeat interviews carried out? If yes, how many?<br>**Four were carried out in total** |
| 19. Audio/visual recording | Did the research use audio or visual recording to collect the data?<br>**Audio recordings were made during each focus group** |
| 20. Field notes | Were field notes made during and/or after the interview or focus group?<br>**No** |
| 21. Duration | What was the duration of the interviews or focus group?<br>**Average length of focus groups = 33 minutes (range = 21-41 minutes)** |
| 22. Data saturation | Was data saturation discussed?<br>**Yes** |
| 23. Transcripts returned | Were transcripts returned to participants for comment |

For peer review only

| | |
|---|---|
| | and/or correction?<br>**No** |
| **Domain 3: analysis and findings** | |
| *Data analysis* | |
| 24. Number of data coders | How many data coders coded the data?<br>**Three** |
| 25. Description of the coding tree | Did authors provide a description of the coding tree?<br>**N/A** |
| 26. Derivation of themes | Were themes identified in advance or derived from the data?<br>**Derived from data** |
| 27. Software | What software, if applicable, was used to manage the data?<br>**NVivo (Version 10, QSR, Southport, UK)** |
| 28. Participant checking | Did participants provide feedback on the findings?<br>**No** |
| *Reporting* | |
| 29. Quotations presented | Were participant quotations presented to illustrate the themes/findings? Was each quotation identified? e.g. participant number<br>**Yes** |
| 30. Data and findings consistent | Was there consistency between the data presented and the findings?<br>**Yes** |
| 31. Clarity of major themes | Were major themes clearly presented in the findings?<br>**Yes** |
| 32. Clarity of minor themes | Is there a description of diverse cases or discussion of minor themes?<br>**No** |

For peer review only

BMJ Open

# Exploring the use of a gamified intervention for encouraging physical activity in adolescents: A qualitative longitudinal study in Northern Ireland

| | |
|---|---|
| Journal: | *BMJ Open* |
| Manuscript ID | bmjopen-2017-019663.R1 |
| Article Type: | Research |
| Date Submitted by the Author: | 16-Feb-2018 |
| Complete List of Authors: | Corepal, Rekesh; Queen's University Belfast, Centre for Public Health<br>Best, Paul; Queen's University Belfast, Centre for Evidence and Social Innovation<br>O'Neill, Roisin; Queen's University Belfast<br>Tully, Mark; Queen's University Belfast,<br>Edwards, Mark; University of Bristol, Centre for Exercise Nutrition and Health Sciences<br>Jago, Russ; University of Bristol, Centre for Exercise, Nutrition and Health Sciences<br>Miller, Sarah; Queen's University Belfast, Centre for Evidence and Social Innovation<br>Kee, Frank; Queen's University Belfast, UKCRC Centre of Excellence for Public Health (NI)<br>Hunter, Ruth; Queen's University Belfast, Centre for Public Health |
| <b>Primary Subject Heading</b>: | Qualitative research |
| Secondary Subject Heading: | Public health |
| Keywords: | PREVENTIVE MEDICINE, PUBLIC HEALTH, QUALITATIVE RESEARCH |
| | |

SCHOLARONE™
Manuscripts

# Exploring the use of a gamified intervention for encouraging physical activity in adolescents: A qualitative longitudinal study in Northern Ireland

Rekesh Corepal, Paul Best, Roisin O'Neill, Mark A. Tully, Mark Edwards, Russell Jago, Sarah Miller, Frank Kee, Ruth F. Hunter

Rekesh Corepal, UKCRC Centre of Excellence for Public Health/Centre for Public Health, Queen's University Belfast, Belfast, Northern Ireland

Paul Best, Centre of Excellence and Social Innovation, School of Social Sciences, Education and Social Work, Queen's University Belfast, Belfast, Northern Ireland

Roisin O'Neill, UKCRC Centre of Excellence for Public Health/Centre for Public Health, Queen's University Belfast, Belfast, Northern Ireland

Mark A. Tully, UKCRC Centre of Excellence for Public Health/Centre for Public Health, Queen's University Belfast, Belfast, Northern Ireland

Mark Edwards, Centre for Exercise Nutrition and Health Sciences, School for Policy Studies, 8 Priory Road, University of Bristol, Bristol, England

Russell Jago, Centre for Exercise Nutrition and Health Sciences, School for Policy Studies, 8 Priory Road, University of Bristol, Bristol, England

Sarah Miller, Centre for Evidence and Social Innovation, School of Social Sciences, Education and Social Work, Queen's University Belfast, Belfast, Northern Ireland

Frank Kee, UKCRC Centre of Excellence for Public Health/Centre for Public Health, Queen's University Belfast, Belfast, Northern Ireland

Ruth F. Hunter, UKCRC Centre of Excellence for Public Health/Centre for Public Health, Queen's University Belfast, Belfast, Northern Ireland

**Corresponding author:** Dr Ruth Hunter, UKCRC Centre of Excellence for Public Health/Centre for Public Health, Institute of Clinical Sciences B, Queen's University Belfast, Belfast, Northern Ireland, BT12 6BJ; E-mail: ruth.hunter@qub.ac.uk; Telephone: +44 (028) 90 978944

**Word count:** 5749

For peer review only

**ABSTRACT**

**Objective:** To explore the temporal changes of adolescents' views and experiences of participating in a gamified intervention to encourage physical activity behaviour, and associated processes of behaviour change.

**Design:** A Qualitative Longitudinal design was adopted whereby focus groups were conducted with the same participants in each intervention school (n=3) at four time-points (baseline, end of each of two intervention phases and 1 year follow-up). The Framework method was used to thematically analyse the data.

**Setting:** Secondary schools (n=3), Belfast (Northern Ireland).

**Participants:** A sub-sample (n=19 at 4 time-points) of 12-14 year olds who participated in the StepSmart Challenge, a gamified intervention involving a pedometer competition and material rewards to encourage physical activity behaviour change.

**Results:** Three core themes were identified: 1) Competition; 2) Incentives and 3) Influence of friends. Participants indicated that a pedometer competition may help initiate physical activity, but suggested that there were a number of barriers such as participants finding it *"boring",* and feeling as though they had a remote chance of *"winning"*. ''Incentives" were viewed favourably, although there were participants who found not winning a prize *"annoying"*. Friends were a motivator to be more physically active, particularly for girls who felt encouraged to walk more when with a friend.

**Conclusions:** The intervention in general and specific gamified elements were generally viewed positively and deemed acceptable. Results suggest that gamification may have an important role to play in encouraging adolescents to engage in physical activity, and in creating interventions that are fun and enjoyable. The longitudinal approach added additional depth to the analysis as themes were refined and tested with participants over time. The findings also suggest that gamified Behaviour Change Techniques align well with core concepts of Self-determination Theory, and that various game-elements may require tailoring for specific populations, for example, different genders.

**Trial Registration Number:** NCT02455986

**Strengths and limitations**

For peer review only

- A major strength of this study was the novel use of a longitudinal design, using the same participants in repeated focus groups. This enabled the investigation of how participants' views, experiences and PA behaviour evolved over time.
- In addition, having a number of researchers involved in the data collection and analyses reduces selectivity and researcher bias.
- However, only three focus groups participated in the study, and all were single sex schools.

For peer review only

**BACKGROUND**

Physical activity (PA) levels in children and adolescents around the world are low. [1] As patterns of PA are established during this time and tend to track into adulthood, [2 3] this is a crucial period which can affect lifelong health and habits. To date, PA programmes for this population have shown limited effect, [4 5] stressing the need for innovative approaches to initiate and maintain PA behaviour. [6]

Programmes such as Pokémon GO illustrates the potential of gamified interventions (i.e. the application of elements of game playing, such as scoring points, competing against others, to change behaviour) for encouraging PA behaviour [7] and can provide useful insights into how to reach and engage the most inactive in PA behaviour. [8] Elements of gamification are incorporated into many commercial PA promotion apps, such as Pokémon GO, Fitocracy and 'Zombies, Run!', which include the collection of points for undertaking a targeted behaviour, completing challenges, or competing against others in virtual games. [9] Interventions that have applied gamification elements suggest it could be possible to make a routine activity such as travelling to school into a game that promotes active travel modes, and that is engaging and fun.[10 11]

Some key gamification strategies, including feedback on players' performance to allow them to set goals and monitor progression, competing with others, and use of incentives, are all evidence-based Behaviour Change Techniques (BCTs).[12] Further, research has demonstrated that other core aspects of gamified interventions such as opportunities for socialisation, self-evaluation, and rewarding positive behaviour are key to providing an enjoyable experience [13 14] and enjoyment has been identified as a significant predictor of PA behaviour.[15] However, gamification interventions have rarely been grounded in well-established theoretical frameworks and we know little about the views and experiences of participants.

The aim of this study was to explore the views and experiences of adolescents who participated in a gamified PA intervention based on Self-determination Theory, and the temporal changes of these views and experiences over the 1-year study period. Study objectives included;

For peer review only

1. To explore key aspects of a gamified PA intervention over a 1-year period using a Qualitative Longitudinal Research method.
2. To discuss key issues relating to the intervention, such as PA opportunities/barriers, the value of competition and types of rewards etc.
3. To explore the key influences of PA and to determine who benefitted from the intervention, how and why it worked for them;
4. To qualitatively chart changes in behaviours, opinions or views as a result of participating in the intervention.

For peer review only

## METHODS

### Context

The StepSmart Challenge was a 24-week primarily school-based intervention utilising team, and individual competitions in five schools in Belfast, Northern Ireland. The school recruitment process is detailed elsewhere (Best et al; under review). An independent trial statistician randomly allocated the five schools to the intervention (three schools) or control (two schools) group. School characteristics are shown in Table 1; two were all-boys schools, two all-girls schools, and one was a co-educational school. All intervention schools were single sex (boys (n=1), girls (n=2)). Students (n=224) from Year 9 classes (aged 12-14 years) were invited to participate in the trial. The main results from the feasibility trial are published elsewhere (Best et al; under review). Briefly, the results demonstrated that the StepSmart Challenge was acceptable to young people for encouraging PA, and there was a trend in increasing light-intensity PA and improving mental wellbeing.

### The StepSmart Challenge

The StepSmart Challenge was a gamified intervention designed according to Self-determination Theory (SDT),[16] using distinct intervention phases aiming to move participants along the motivation continuum from extrinsic motivation towards intrinsic motivation and encouraging PA behaviour change. The theory is grounded in three psychological needs – autonomy, competence, and relatedness. [16] Those intrinsically motivated engage in PA for the enjoyment and satisfaction it provides. [17] This form of motivation is associated with improved quality of life, increased PA behaviour, [18] and long-term behaviour change. [19] Self-motivation is undermined when individuals feel less control over the activity, and the

environment, and if they do not feel a sense of connectedness or belonging to others engaging in the same activity. [20]

Table 2 details the various intervention components and links to BCTs. The intervention consisted of two phases. Phase One involved a multi-level (competition at the school, team and individual level) pedometer competition lasting for 8-weeks. Team selection was determined by the research team and took account of current PA levels and friendship networks measured at baseline; this was to ensure a mixed ability team (4-5 participants per team) with at least one friend in the team. The team competition entailed social incentives such as publication of the results on the website, a trophy awarded to the leading team (i.e. the team with the highest number of total steps) in each school at competition end, and a prize to the winning school (£1000). During the individual competition in Phase One, incentives (approximate value of £10 – see Table 2 for details) were awarded weekly in each school to two participants (participant who accumulated the most steps that week ('Walker of the Week'); and the participant who increased his/her step count the most from the previous week ('Most Improved')). Phase Two (14 weeks) focused on an individual level competition, in which the three participants that had accumulated the most steps in each school during this phase were awarded a 'goody bag' (approximate value of £30 for each participant and consisted of an assortment of those used in Phase One).

**Qualitative longitudinal research**

To elicit the temporal views and experiences of participants in the StepSmart Challenge a qualitative longitudinal research (QLR) design was used involving repeated semi-structured focus groups with the same participants over four data collection periods (recurrent cross-sectional approach). This enabled qualitative charting of the perceived behaviour change as well as changes in attitudes and opinions over a one-year period. This presented an opportunity to further understand potential mechanisms of behaviour change, and how perceptions and experiences of the intervention changed over time (pre, during and post-intervention). [21] Understanding why certain choices were made can produce more insightful and considered interpretation of behaviour change. [22] Such approaches are particularly valuable in providing a different perspective in assessing interventions or as part of process evaluations. [23]

**Focus group participants**

For peer review only

Baseline focus groups were conducted in each intervention school with a sub-sample of trial participants using a purposive sampling strategy whereby teachers identified potential participants with a range of PA levels from low-to-high as well as those with mixed educational ability. To reduce selection bias, the researchers discussed the importance of having a range of views within focus groups before participants were selected. However, it was considered that teachers were best placed to make these judgements as researchers did not know any of the participant's backgrounds and would not be aware of hidden conflicts or instances of bullying which may have influenced the group dynamic and quality of data. Those interested in taking part were given a study information sheet by the teacher explaining the purpose of the focus groups, to read themselves and then give to their parent(s)/guardian(s). Parental (or guardian) opt-out consent and participant consent was sought from all participants. [24] Ethical approval was granted by the School of Medicine, Dentistry and Biomedical Science Research Ethics Committee (Queen's University, Belfast) (Ref: 15.09).

The focus groups were repeated during the study, following the same participants on their journey through the trial. This provided rich contextual data to explore the views and experiences of participants over time. Data were collected one-month pre-intervention (T0); at the end of the team competition (8 weeks) (T1); end of the individual competition (post-intervention) (24 weeks) (T2); and at 12-month follow-up (T3). Focus groups were conducted on school premises and were audio recorded. The researchers verbally reaffirmed consent to participate at the beginning of each focus group. No other participants were present at the time of the focus groups.

Focus groups were semi-structured, based on topic guides (see Supplementary Material I) exploring core concepts at each time point. The topic guide was not piloted but developed iteratively reflecting on the data gathered from the focus groups from previous time points. Thus, emerging themes were explored across time points to chart changing views, experiences and PA behaviour. During all focus groups, the researcher summarised information at the end of each section and questioned understanding as a form of participant verification. [25]

Core concepts explored included:

1. General views and experiences of the intervention and intervention components;

For peer review only

2. Motivation to be active and to sustain activity long-term;
3. Extrinsic motivators including a) competition e.g. Does the competition motivate you to walk?; b) Material incentives e.g. Was the opportunity to win a prize something that motivated you?; c) Motivation for PA e.g. What motivates you to be active?;
4. SDT concepts including a) Autonomy e.g. What new ways have you found to be active?; b) Perceived competence e.g. How did it make you feel when you compared your steps to those of the class?; c) Relatedness e.g. Do you think friends are important in terms of how active you are?

Focus groups were conducted by RC (Male, PhD student), PB (Male, Post-doctoral Researcher) and RO'N (Female, Post-doctoral Researcher). PB and RO'N are experienced qualitative researchers and have facilitated focus groups with adolescents previously. RC had undergone a number of formal training courses in the facilitation of focus groups and thematic analysis methods. RC was accompanied to the focus groups by either RO'N or PB. Saturation of the data was discussed between PB and RC. None of the researchers had any relationship with the participants.

**Data analysis**

Focus group recordings were transcribed verbatim and anonymised. Data were imported into NVivo (Version 10, QSR, Southport, UK) to manage and analyse the transcripts. Analysis was undertaken using the Thematic Analysis Framework at the semantic level using a recurrent cross-sectional approach. [26] Initially researchers (RC and PB) familiarised themselves with the data. A sample coding frame was developed by the researchers independently, and refined iteratively with subsequent discussions. As a result, three coding frameworks were generated, one for each core theme. Illustrative quotes supporting emerging themes were highlighted and agreed by researchers. Transcripts were not returned to participants for comment, and they did not provided feedback on findings.

The dataset was systematically coded using an inductive approach and codes were generated to give a summary of elements of analytic interest. Once coded researchers identified potential themes from clusters of codes of similar meaning as well as patterns of responses across codes. Three central themes were identified at T0 and developed at subsequent time points. The coding frame was then discussed with ME and further refined. RC and ME then met multiple times to discuss and refine codes until a final coding frame

For peer review only

was applied to all data. These themes consisted of (1) incentives; (2) competition; (3) influence of friends on PA. The temporal changes in the views and experiences of participants, and the influence of these components on the process of behaviour change were explored under each theme. Researchers (RC and ME) analysed the data together to further refine sub-themes to ensure assertions were accurately reflected. Codes were not validated with study participants due to the time commitment that they had already provided due to the qualitative longitudinal design. However, given the nature of the QLR design core concepts that were apparent at T0 were revisited at subsequent time points to test the validity of the theory.

**RESULTS**

Table 3 details the characteristics of participants in the focus groups, and demonstrates that the purposive sampling strategy was successful in recruiting participants of mixed gender, SES, from different teams, those who won prizes and those who didn't win, and PA levels. Table 3 displays a breakdown of the focus groups conducted. Twelve focus groups were conducted (mean duration 33 minutes; range 21–41 minutes (Table 4)). Focus group participants present at each time point varied due to participant absences (mean 6 participants; range 2–7 participants). No participants refused to participate or dropped out.

The following results section details the themes and sub-themes identified from the thematic analysis. This includes a diagrammatic representation (see figure 1) of how themes continued to evolve as new data emerged at each time point.

**Theme 1: Competition**

Three sub-themes emerged under this theme: a) usefulness of competition for PA behaviour change; b) self-competition for PA behaviour change; c) experiences of the team and individual competition.

*Sub-theme A: Usefulness of competition for PA behaviour change*

At T0, participants suggested the competition would motivate them to work harder and it was generally viewed as a motivating factor to become more physically active:

*"that's what motivates me"* (School C, Male 5, T0).

*"that's what pushes people on"* (School C, Male 6, T0).

For peer review only

*"if I was actually in a competition I'd actually walk everywhere"* (School D, Female 6, T0).

As interviews progressed (across time points) these early conceptualisations were developed further. For example, it was clear from T1 onwards that for a sub-sample of participants in all schools the desire to compete was not sufficient; the goal of winning was paramount:

*"we like the winning but we don't like the losing"* (School E, Female 3, T0).

*"to try and win like every week after that"* (School C, Male 6, T3).

*"yeah, because you just wanted to win"* (School D, Female 6, T3).

Yet while this sub-sample was extremely motivated during Phase 1, when the competitive elements (against others) lessened during Phase 2, the intervention became monotonous or 'boring':

*"I think that's boring you know, who would want to know how many steps you're taking?"* (School D, Female 2, T2).

*"at the start like, like you were quite motivated and then it just got more on and then you just forgot to wear it some days and it just got quite boring"* (School C, Male 4, T3).

*"It was just like the same thing every day"* (School C, Male 4, T3).

For others, the benefits of competition related to their perceived their chances of winning. If this was believed to remote then motivation lessened. This became clear at T1 as the researcher reflected upon earlier (T0) responses given by participants.

*"whenever you found out that you're actually losing there's just no point"* (School D, Female 2, T1).

*"it's just sort of cause you knew you probably weren't going to win so you're just like there's really no point in wearing it [pedometer]"* (School D, Female 6, T2).

*"well I just really gave up whenever X just won everything. I really did. I just stopped"* (School D, Female 3, T3).

For peer review only

*Sub-theme B: Perceptions of the usefulness of self-competition for PA behaviour change*

The potential use of the pedometer for self-directed goals, or 'self-competition' (competing against oneself) was considered promising at T0:

> *"if its showing you how many steps you're taking then you could challenge yourself to take more every day. So if you took 2000 steps one day you could try like try take more the next day. So it's like challenging yourself"* (School D, Female 1, T0).

Self-competition was shown to be a motivating factor throughout the intervention for most participants. One participant (School D) viewed 'getting better' and improving step counts as meaningful, reducing the negative effect of not winning prizes. This aligns closely with SDT and shows possible progression towards intrinsically based motivated for PA.

> *"trying to beat your day before target"* (School C, Male 5, T1).
> *"I loved to see like how many steps you were actually taking like when you're beating your scores as well like you check it one day and then the next day your gonna try and beat it"* (School E, Female 1, T2).
> *"No, it's alright because I was getting more each day so I was getting better; so it was alright"* (School D, Female 6, T3).

*Sub-theme C: Experiences of the team and individual competition*

The intervention incorporated two formats of a pedometer competition: a team-based competition (8 weeks duration), and an individual competition (14 weeks duration). During the individual competition, participants competed against others from their school. At T0 the team-based competitions were seen to have the potential to better encourage PA:

> *"you can work together as a team"* (School D, Female 4, T0).

For peer review only

*"if you were in like a group with more active people like you'd be sad that you're not as good as them but it would kind of push you to be as good as them"* (School E, Female 7, T0).

When asked to compare the individual with the team-based competition at T2, many participants from School C (all male) were more motivated by the team-based competition. This was due to the support provided by the team or peer pressure from not wanting to let *"your team down"*:

*"I'd probably say it's easier with the support rather than individually"* (School C, Male 6, T2).

*"you didn't want to let your team down"* (School C, Male 6, T2).

Reflecting on the intervention at T3, there were clear differences regarding experiences of the competition. School C (all boys) participants continued to feel positive about the team competition.

*"the team's a lot better. Like its more… you are just like together"* (School C, Male 1, T3).

*"The individual is quite boring"* (School C, Male 4, T3).

The girls' schools (School D and E) tended to favour the individual competition, as they did not have to rely on their team members, or for logistical reasons, such as the inability to meet up and 'organise stuff'.

*"you don't have to depend on anyone else"* (School D, Female 3, T3).

*"you know like sometimes you don't live close to your friends so you can't always organise stuff, which is a problem. So I think the individual one"* (School E; Female 7, T3).

One disadvantage of the team competition was the issue of free-riding team members (i.e. a member of a team that obtains benefits from membership but is not seen to contribute a fair share of the work needed to accrue the benefit). [27] In School D (all girls), free-riding was evident.

For peer review only

> *"well in the group you didn't really have to do anything cause the rest of them could do it but like by yourself like I don't know you just lose it altogether cause you don't walk"* (School D, Female 2, T2).
>
> *"we didn't really have to worry about it cause like the rest of them would've like walked anyway"* (School D, Female 2, T2).

**Theme 2: Incentives**

Two sub-themes were identified including a) type of incentive; and, b) perceptions of usefulness on incentives.

*Sub-theme A: Type of Incentive*

The provision of material incentives in this study was contingent on doing well in the competition, rather than being contingent on PA behaviour change. The types of incentives suggested and discussed by participants included recognition-based incentives (e.g. trophies) and material incentives (e.g. vouchers). Males tended to favour recognition based incentives, whereas material incentives with a higher monetary value were largely proposed by females. While this was apparent at T0, the QLR approach enabled the researchers to revisit this at subsequent time points to test the validity of the theory. When asked what type of prizes they would like, male participants suggested:

> *"a medal or a trophy"* (School C, Male 5, T0).
>
> *"rugby ball"* (School C, Male 4, T0).

In contrast, females often suggested the use of material incentives.

> *"vouchers for clothes"* (School D, Female 6, T0).
>
> *"Topshop"* (School D, Female 1, T0).
>
> *"money"* (School E, Female 7, T0).

*Sub-theme B: Perceptions of usefulness of incentives*

At T1 and T2 many participants viewed incentives favourably. Participants suggested that the incentives were desirable and encouraged them more during the intervention.

For peer review only

*"Every week cos you know it's like running out of time for like the prizes, just really want to get one"* (School C, Male 6, T1).

*"that they weren't just like wee rubbish prizes they were really good ones"* (School E, Female 4, T2).

At T3, when reflecting on the intervention, participants still viewed the incentives as a motivating factor as they were 'good' prizes and provided acknowledgement for achievement:

*"yeah, they look good. Like the prizes were really good"* (School C, Male 4, T3).

*"yeah, I think [it] was good actually. Just to keep people motivated* (School E, Female 7, T3).

*"like you know you are being acknowledged, like when you get prizes"* (School E, Female 3, T3).

Some participants did not win any prizes over the course of the intervention. When these participants discussed the instances when their peers won prizes there was a clear sense of disappointment, with a number stating that it was *"annoying":*

*"kinda annoyed you when people like brought out their ten pound of cinema tickets and yeah it's like kinda annoying"* (School C, Male 3, T2).

*"like it annoyed me that I didn't get one"* (School E, Female 6, T2).

*"It just made me sad"* (School E; Female 2, T2).

**Theme 3: Influence of friends**

Two sub-themes emerged, including: a) the role of friends in general for encouraging PA behaviour, and b) the role of friends in team competition.

*Sub-theme A: Role of friends in general for encouraging PA behaviour*

At T0, participants suggested that PA was more enjoyable with friends, and the social support provided by friends encouraged participation in PA:

For peer review only

> *"it's about encouraging each other to do stuff"* (School C, Male 5, T0).
>
> *"if they want to go for a run you will want to go for a run with them"* (School C, Male 4, T0).
>
> *"Yeah, because you want to be doing it with them so you can enjoy yourself"* (School C, Male 6, T3)
>
> *"Good friends will help you yeah"* (School D, Female 6, T0).
>
> *"X only lives up the street so we go for runs most days after school"* (School E, Female 3, T0).

Participants in School E discussed the continued positive influence of friends on PA motivation at T1 and T2. This added additional depth to T0 findings by showing friends as providing a social acceptable context in which to be active. Feeling 'scundered' (colloquialism for embarrassed) when walking alone is offset when provided the social support of friends:

> *"Make you feel like I'm going to be scundered [embarrassed] walking about alone but when you have your friend with you like you'd be more encouraged to do more walking if you're like walking with your friend"* (School E, Female 2, T2).
>
> *"hardly just like go a walk about yourself about the street like a big loner"* (School E, Female 4, T2).

*Sub-theme B: Role of friends in team competition*

Participants suggested that the influence of friends and a sense of connectedness was necessary within teams in order for them to work together and be competitive. Although, some participants in School C felt it would be *"good to have at least one friend or two"* (School C, Male 6, T3), they did not want to pick their own teams as they believed this might produce imbalanced teams with the more physically active individuals going into the same team. For these participants, the combination of friends within a team and homogeneity between teams was important:

> *"No cause then they could get really unfair"* (School C, Male 1, T2).

For peer review only

*"Cause all the active people could go in one team and then the inactive so it wouldn't work out"* (School C, Male 1, T2).

In contrast, participants in Schools D and E wanted the opportunity to choose team members; preferring to be in teams composed of their friends:

*"I wanted to choose my own team"* (School D, Female 4, T1)
*"no I think it should just be like your own group like friends like five of each of them"* (School E, Female 3, T1).
*"because like [if] you don't like people in your team you're just going to be like 'nah not even going to talk to you'"* (School E, Female 6, T2).

Figure 1 here

Figure 1 illustrates the various thematic pathways that developed during focus group interviews at T0 (red), T1 (yellow), T2 (orange) and T3 (green). Taking the first theme (competition) as an example, the researchers considered the emergence of two distinct groups at T0. These were (1) *physically active participants* who viewed a pedometer competition as a means of further increasing their active lifestyle and, (2) *less active participants* who viewed the pedometer competition as an opportunity to become more active.

At T0, physically active participants were particularly 'excited' about a pedometer-based competition, perceiving it as enjoyable process. Some of these participants appeared to engage regularly in competitions. The less active group were more cautious, but felt a pedometer based competition might provide an acceptable context through which PA may be enjoyable. As such, these early ideas/themes were represented at T0 (red).

As interviews progressed through T1 and T2, the research team observed changes in relation to participant's views of competition (both in a general sense as well as relating directly to StepSmart). Moreover the QLR approach enabled the research team to frame these changes within the context of data revealed at baseline (T0). For example, physically active members who regularly engaged in competitions (outside of the StepSmart Project) at T0 continued to enjoy the intervention at T1 and T2. However, for a sub-set of this group (where winning

For peer review only

was more important than competing) motivation lessened at T2. Figure 1 also illustrates that for participants who were less active, motivation and engagement decreased much sooner (at T1) and continued to decrease into T2 and T3. In some cases these participants appeared to have been motivated solely by the material incentives, thus the perceived failure of 'not winning a prize' was interpreted as negative feedback and reinforced negative schemas around PA.

Self-determination theory maintains that an activity that is stimulating is an important aspect of sustained motivation. Throughout the intervention, the importance of self-monitoring, and the importance of personal goals was prominent for all participants. The concept of self-competition provides an opportunity to challenge oneself and can be supportive of feelings of competence. Self-competition, provided an opportunity for all participants to receive positive feedback by meeting the goals they set for themselves, and could lessen the impact of not winning prizes. By adopting a QLR approach, themes generated in earlier focus groups evolved and could be tested as new data emerged. The same level of depth would have been difficult to achieve within a pre-post test design.

**DISCUSSION**

Participant's generally had positive experiences and views of this gamified PA intervention. Results suggested that the gamified design may have had an important role to play in encouraging adolescents to engage in PA, and in creating interventions that are fun and enjoyable. The findings also suggested that core concepts of SDT are compatible with gamified BCTs, and that some game elements may require tailoring for specific populations, for example, different genders.

In general, the use of a gamified pedometer competition was viewed favourably by participants. The goal of winning was very important for some and was key to sustaining their motivation to be active. This could be linked to the provision of material incentives which was contingent on 'winning' the competition. Over the course of the intervention, material incentives continued to motivate some participants. A possible mechanism could be that positive feedback provided by winning prizes and doing well in the competition helped develop an individual's intrinsic motivation by improving feelings of competence. [28] The positive effect of material incentives for health behaviour change with children and adolescents has also been shown in previous studies. [29-32]

For peer review only

The long-term effect is less clear with some studies showing that positive effects dissipate over time. [33] This could be due to habituation to the extrinsic motivators being offered [34] or a 'crowding out effect' of intrinsic motivation [35] once extrinsic incentives are removed. However, to date this hypothesis has not been tested or supported in 'real world' interventions. [28 36-38]

Other participants felt de-motivated from the outset as they believed they had no chance of winning. Some became less enthusiastic about the competition if other participants consistently had a greater number of steps, and were disappointed at not winning a prize. Previous work has suggested that competition can affect participants' self-evaluation of their competence to perform the task. [39 40] If a participant loses, and their loss is attributed to low ability, this can negatively impact behaviour. [41] Therefore, participants may choose not to compete, or not engage in the competition with maximum effort. [42] This helps to provide some explanation for the loss, other than low ability, thus preserving the participant's self-esteem and self-efficacy.

Some participants indicated that they became gradually less interested because of the repetitive nature of the pedometer competition. These findings are supported by a large body of literature which suggests that extrinsic motivators can have a short-term positive effect on motivation which is not maintained. [33 36 37 43] Extrinsic motivators such as competition and material incentives could be used to initially stimulate the interest of participants, especially those with lower levels of PA. [44] However, a key learning point would be to transition to more intrinsically motivating forms of PA and thus the incorporation of BCTs that focus on these behaviours would be useful. [45 46]

The competition had various levels: rewards could be offered to the highest achieving team, the highest achieving individual, or to anyone on the basis of achieving some personal goals (self incentive). The findings showed distinct perceptions regarding the value of each. For example, males tended to prefer the team competition and suggested they would try harder to contribute to the team, and found the team environment supportive and enjoyable. Maculada [47] suggested that males find team affiliation important, and a way to be accepted by peers and to feel a sense of belonging with the group. Team-based PA interventions have been shown to be effective, [10 11] and may be less harmful than individual competitions. [48]

For peer review only

Conversely, females favoured individual competition; how well a participant did in the competition was not dependent on the effort of others, mitigating to the problem of free-riding. [49] One solution may be to distribute incentives equitably (ie proportionate to effort and contribution) to team members rather than distributing them equally, [50] thereby reducing free-riding and increasing effort. [51 52]

Self-competition [53] was seen as a prominent positive influence of PA. Participants often used the pedometers for feedback, to self-monitor, and set personal step goals. Creating achievable personal goals may also play a part in mitigating the potential negative effects of extrinsic motivators by emphasising competence (by meeting goals and receiving positive feedback), autonomy (as participants are free to choose which activities they pursue to increase step counts), and maintaining self-efficacy. Self-competition with the use of intrinsic goals was enjoyed by all participants in the focus groups, regardless of success in the overall competition. Self-competition allowed participants to be autonomous, and to create achievable challenges such as walking more steps than during the previous day. Therefore, self-competition could be a way to develop autonomous identified or integrated regulation, which has been shown to have benefits for PA motivation. [54 55] Autonomy-supportive elements such as self-competition could consequently stimulate the development of habit formation. [56]

The significance of friends for influencing PA behaviour has also been frequently cited in the literature. [57-59] The participants' feelings on team composition and the influence of friends reinforce the psychological need for relatedness, a core construct of SDT. Participants from all schools felt that a sense of connectedness to the group was important for an effective team competition. Other research shows that adolescents value opportunities for social interaction, [39] and so team membership could have a positive effect on PA motivation. Participants stated that friends provided support, encouragement and help with the enjoyment of PA.

**Reflections on the QLR approach**

The authors acknowledge the difficulty in mapping temporal changes, especially in focus groups, where there may not be sufficient time or opportunity to explore individual's views in detail. Nonetheless, Figure 1 is a simplified, but useful, thematic illustration of general (group-level) consensus over a 1 year period.

For peer review only

The complexities contained within each pathway highlight the difficulty in developing a group-based PA intervention that will motivate all participants in a similar manner. It also illustrates the interrelated nature of the themes, and how experiences of one aspect of the intervention can influence other components. However, recognition-based incentives, the provision of feedback on performance, and opportunities for social connectedness were shown to be key gamification strategies with potential for motivating PA throughout the intervention period. This is in line with SDT which posits that supporting innate desires, competence and a sense of relatedness with others could help achieve a higher quality of motivation that is long-lasting.

**Strengths and limitations**

A major strength of this study was the novel use of a longitudinal design, [21 60] using the same participants in repeated focus groups at baseline, post-intervention and 1-year follow-up. This enabled the study of how participants' views, experiences and PA behaviour evolved over time. The findings are robust as assumptions, views and experiences can be tested and re-tested in subsequent sessions, and researchers build relationships with participants due to the repeated exposure which can encourage disclosure. In addition, having a number of researchers involved in the data collection and analyses reduces selectivity and researcher bias.

A focus group method was chosen as it provided an opportunity for the group to discuss issues amongst themselves and reach consensus, gathering multiple viewpoints and representing 'everyday' conversation. However, the approach has been criticised for lacking depth, particularly when conducted with young people as they tend not to elaborate on discussion points. It may also have been useful to combine this approach with 1:1 interviews to reduce peer pressure and ensure coherence of responses at different schools. Only three focus groups participated in the study, and all were single sex schools. Consequently the purported gender differences may be an artefact of differences in socio-economic status as well as or in addition to gender differences. Finally, there was good retention of participants in the qualitative longitudinal design, with the exception of T3 in which four (out of six) pupils were missing from School D owing to a timetable clash that was beyond the control of the research team.

For peer review only

**Conclusions**

Preferences for gamified elements including team or individual competitions, and the influence of friends on PA behaviour were highlighted. The use of a longitudinal qualitative design enabled exploration of temporal changes in participants' views and experiences, and exploration of potential mechanisms of behaviour change. This study suggests that the three core constructs for self-motivation in SDT could be important factors for motivating PA in adolescents via competition and the use of material rewards delivered through gamification. This supports previous research which proposes benefits in providing opportunities for autonomy, perceived competence, and relatedness. [61]

**Acknowledgments:** The authors wish to thank the pupils and teachers who were involved in the development of this intervention, and those who participated in the study.

**Competing Interests:** The authors declare that they have no competing interests.

**Data Sharing Statement:** No additional data are available.

**Contributions Statement:** RH had the initial idea for the study and led the writing of the grant with significant contribution from MT and FK. RH, PB, MT and FK were involved in the design of the study. RC, PB and RON facilitated the focus group discussions. RC, PB, RON and ME were involved in the analysis of the data. All authors (RC, PB, RON, MT, ME, RJ, SM, FK and RH) were involved in the interpretation of the data. All authors (RC, PB, RON, MT, ME, RJ, SM, FK and RH) provided substantial comments on the drafts of the manuscript and approved the final version.

**Funding:** The authors wish to acknowledge funding from a HSC R&D (NI) Enabling Research Award. RH is supported by a NIHR Career Development Fellowship and acknowledges funding support from the HSC Research and Development Division. The work was undertaken under the auspices of the UKCRC Centre of Excellence for Public Health Research Northern Ireland and Centre for Diet and Activity Research (CEDAR), UKCRC Public Health Research Centres of Excellence which are funded by the British Heart Foundation, Cancer Research UK, Economic and Social Research Council, Medical Research Council, the National Institute for Health Research, and the Wellcome Trust. The funders had no role in study design, data collection, data analysis, data interpretation, or writing of the report.

For peer review only

**References**

1. Tremblay MS, Barnes JD, González SA, et al. Global Matrix 2.0: Report card grades on the physical activity of children and youth comparing 38 countries. *J Phys Act Health* 2016;13(11 Suppl 2):S343-S66. doi: 10.1123/jpah.2016-0594

2. Telama R. Tracking of physical activity from childhood to adulthood: a review. *Obes Facts* 2009;2(3):187-95. doi: 10.1159/000222244

3. Telama R, Yang X, Leskinen E, et al. Tracking of physical activity from early childhood through youth into adulthood. *Medicine and science in sports and exercise* 2014;46(5):955-62. doi: 10.1249/MSS.0000000000000181

4. Metcalf B, Henley W, Wilkin T. Effectiveness of intervention on physical activity of children: systematic review and meta-analysis of controlled trials with objectively measured outcomes (EarlyBird 54). *BMJ (Clinical research ed)* 2012;345:e5888.

5. Soares NM, Leão AS, Santos JR, et al. Systematic review shows only few reliable studies of physical activity intervention in adolescents. *ScientificWorldJournal* 2014;2014:206478. doi: 10.1155/2014/206478

6. Gillis L, Tomkinson G, Olds T, et al. Research priorities for child and adolescent physical activity and sedentary behaviours: an international perspective using a twin-panel Delphi procedure. *The international journal of behavioral nutrition and physical activity* 2013;10:112. doi: 10.1186/1479-5868-10-112

7. McCartney M. Margaret McCartney: Game on for Pokémon Go. *BMJ (Clinical research ed)* 2016;354:i4306.

8. Wong FY. Erratum to: Influence of Pokémon Go on physical activity levels of university players: a cross-sectional study. *Int J Health Geogr* 2017;16(1):17. doi: 10.1186/s12942-017-0089-5

9. Lister C, West JH, Cannon B, et al. Just a fad? Gamification in health and fitness apps. *JMIR Serious Games* 2014;2(2):e9. doi: 10.2196/games.3413

10. Hunter RF, de Silva D, Reynolds V, et al. International inter-school competition to encourage children to walk to school: a mixed methods feasibility study. *BMC research notes* 2015;8:19. doi: 10.1186/s13104-014-0959-x

11. Coombes E, Jones A. Gamification of active travel to school: A pilot evaluation of the Beat the Street physical activity intervention. *Health Place* 2016;39:62-9. doi: 10.1016/j.healthplace.2016.03.001

12. Michie S, Richardson M, Johnston M, et al. The behavior change technique taxonomy (v1) of 93 hierarchically clustered techniques: building an international consensus for the reporting of behavior change interventions. *Ann Behav Med* 2013;46(1):81-95. doi: 10.1007/s12160-013-9486-6

13. Sardi L, Idri A, Fernandez-Aleman JL. A systematic review of gamification in e-Health. *Journal of biomedical informatics* 2017;71:31-48. doi: 10.1016/j.jbi.2017.05.011

14. Cugelman B. Gamification: what it is and why it matters to digital health behavior change developers. *JMIR Serious Games* 2013;1(1):e3. doi: 10.2196/games.3139 [published

15. Best P, Tully MA, Corepal R, et al. Time to 're-think' physical activity promotion for young people? Results from a repeated cross-sectional study. *BMC Public Health* 2017;17(1):208. doi: 10.1186/s12889-017-4136-8

16. Ryan RM, Deci EL. Self-determination theory and the facilitation of intrinsic motivation, social development, and well-being. *The American psychologist* 2000;55(1):68-78.

17. Ryan RM, Williams GC, Patrick H, et al. Self-determination theory and physical activity: The dynamics of motivation in development and wellness. *Hellenic Journal of Psychology* 2009;6(2):107-24.

18. Ryan RM, Patrick H, Deci EL, et al. Facilitating health behaviour change and its maintenance: Interventions based on self-determination theory. *European Health Psychologist* 2008;10(1):2-5.

19. Silva MN, Marques MM, Teixeira PJ. Testing theory in practice: The example of self-determination theory-based interventions. *Eur Health Psychol* 2014;16(5):171-80.

20. Sebire SJ, Kesten JM, Edwards MJ, et al. Using self-determination theory to promote adolescent girls' physical activity: Exploring the theoretical fidelity of the Bristol Girls Dance Project. *Psychol Sport Exerc* 2016;24:100-10. doi: 10.1016/j.psychsport.2016.01.009

21. Saldana J. Longitudinal Qualitative Research: Analysing change through time. California: AltaMira Press 2003.

22. Corden A, Millar J. Qualitative longitudinal research for social policy - Introduction to themed section. *Social Policy and Society* 2007;6(4):529-32.

23. Calman L, Brunton L, Molassiotis A. Developing longitudinal qualitative designs: lessons learned and recommendations for health services research. *BMC Med Res Methodol* 2013;13:14. doi: 10.1186/1471-2288-13-14

24. Jago R, Bailey R. Ethics and paediatric exercise science: issues and making a submission to a local ethics and research committee. *J Sports Sci* 2001;19(7):527-35. doi: 10.1080/026404101750238980

25. Harper M, Cole P. Member checking: can benefits be gained similar to group therapy? *The Qualitative Report* 2012;17(2):510-17.

26. Braun V, Clarke V. Using thematic analysis in psychology. *Qualitative Research in Psychology* 2006;3(2):77-101.

27. Albanese RvF, David D. Rational behavior in groups: The free-riding tendency. *Academy of Management Review* 1985;10(2):244-55. doi: 10.5465/AMR.1985.4278118

28. Barte JC, Wendel-Vos GC. A systematic review of financial incentives for physical activity: The effects on physical activity and related outcomes. *Behav Med* 2015:1-12. doi: 10.1080/08964289.2015.1074880

For peer review only

29. Suchert V, Isensee B, Sargent J, et al. Prospective effects of pedometer use and class competitions on physical activity in youth: A cluster-randomized controlled trial. *Prev Med* 2015;81:399-404. doi: 10.1016/j.ypmed.2015.10.002

30. Hardman CA, Horne PJ, Fergus Lowe C. Effects of rewards, peer-modelling and pedometer targets on children's physical activity: a school-based intervention study. *Psychology & health* 2011;26(1):3-21. doi: 10.1080/08870440903318119

31. Horne PJ, Hardman CA, Lowe CF, et al. Increasing children's physical activity: a peer modelling, rewards and pedometer-based intervention. *Eur J Clin Nutr* 2009;63(2):191-8. doi: 10.1038/sj.ejcn.1602915

32. Finkelstein EA, Tan YT, Malhotra R, et al. A cluster randomized controlled trial of an incentive-based outdoor physical activity program. *J Pediatr* 2013;163(1):167-72.e1. doi: 10.1016/j.jpeds.2013.01.009

33. Cahill K, Hartmann-Boyce J, Perera R. Incentives for smoking cessation. *Cochrane Database Syst Rev* 2015(5):CD004307. doi: 10.1002/14651858.CD004307.pub5

34. Burns RJ, Donovan AS, Ackermann RT, et al. A theoretically grounded systematic review of material incentives for weight loss: implications for interventions. *Ann Behav Med* 2012;44(3):375-88. doi: 10.1007/s12160-012-9403-4

35. Frey BS, Jegen R. Motivation Crowding Theory. *Journal of Economic Surveys* 2001;15(5):589-611. doi: 10.1111/1467-6419.00150

36. Mantzari E, Vogt F, Shemilt I, et al. Personal financial incentives for changing habitual health-related behaviors: A systematic review and meta-analysis. *Prev Med* 2015;75:75-85. doi: 10.1016/j.ypmed.2015.03.001

37. Mitchell MS, Goodman JM, Alter DA, et al. Financial incentives for exercise adherence in adults: systematic review and meta-analysis. *Am J Prev Med* 2013;45(5):658-67. doi: 10.1016/j.amepre.2013.06.017

38. Strohacker K, Galarraga O, Williams DM. The impact of incentives on exercise behavior: a systematic review of randomized controlled trials. *Ann Behav Med* 2014;48(1):92-9. doi: 10.1007/s12160-013-9577-4

39. Knowles AM, Niven A, Fawkner S. A qualitative examination of factors related to the decrease in physical activity behavior in adolescent girls during the transition from primary to secondary school. *J Phys Act Health* 2011;8(8):1084-91.

40. Ames C. Achievement attributions and self-instructions under competitive and individualistic goal structures. *Journal of Educational Psychology* 1984;76(3):478-87.

41. Lam SF, Yim PS, Law JS, et al. The effects of competition on achievement motivation in Chinese classrooms. *Br J Educ Psychol* 2004;74(Pt 2):281-96. doi: 10.1348/000709904773839888

42. Grant D, Green W. Grades as incentives. *Empirical Economics* 2013;44(3):1563-92.

For peer review only

43. Giles EL, Robalino S, McColl E, et al. The effectiveness of financial incentives for health behaviour change: systematic review and meta-analysis. *PLoS One* 2014;9(3):e90347. doi: 10.1371/journal.pone.0090347

44. Hunter RF, Tully MA, Davis M, et al. Physical activity loyalty cards for behavior change: a quasi-experimental study. *Am J Prev Med* 2013;45(1):56-63. doi: 10.1016/j.amepre.2013.02.022

45. Seghers J, Vissers N, Rutten C, et al. Intrinsic goals for leisure-time physical activity predict children's daily step counts through autonomous motivation. *Psychology of Sport and Exercise* 2014;15(3):247-54. doi: 10.1016/j.psychsport.2014.01.003

46. Sebire SJ, Jago R, Fox KR, et al. Testing a self-determination theory model of children's physical activity motivation: a cross-sectional study. *The international journal of behavioral nutrition and physical activity* 2013;10:111. doi: 10.1186/1479-5868-10-111

47. Maculada ST. Gender differences in task and ego goal orientation and motivation in sport participation. *Philippine Journal of Psychology* 2007;40(1)

48. Cantador I, Conde JM. Effects of competition in education: A case study in an e-learning environment. Proceedings of the IADIS International Conference E-learning; 2010.

49. Maiden B, Perry B. Dealing with free‐riders in assessed group work: results from a study at a UK university. *Assessment & Evaluation in Higher Education* 2011;36(4):451-64.

50. Garbers Y, Konradt U. The effect of financial incentives on performance: A quantitative review of individual and team‐based financial incentives. *Journal of Occupational and Organizational Psychology* 2013;87(1):102-37. doi: 10.1111/joop.12039

51. Gunnthorsdottir A, Rapoport A. Embedding social dilemmas in intergroup competition reduces free-riding. *Organizational Behavior and Human Decision Processes* 2006;101(2):184-99.

52. Kugler T, Rapoport A, Pazy A. Public good provision in inter‐team conflicts: Effects of asymmetry and profit‐sharing rule. *Journal of Behavioral Decision Making* 2010;23(4):421-38.

53. Howe LA. On competing against oneself, Or 'I need to get a different voice in my head'. *Sport, ethics and Philosophy* 2008;2(3):353-66. doi: Sports Ethics and Philosophy, Vol. 2, No. 3, December 2008, pp. 353–366

54. Van den Berghe L, Vansteenkiste M, Cardon G, et al. Research on self-determination in physical education: key findings and proposals for future research. *Physical Education and Sport Pedagogy* 2014;19(1):91-121. doi: info:doi/10.1080/17408989.2012.732563

55. Teixeira PJ, Carraça EV, Markland D, et al. Exercise, physical activity, and self-determination theory: a systematic review. *The international journal of behavioral nutrition and physical activity* 2012;9:78. doi: 10.1186/1479-5868-9-78

For peer review only

56. Gardner B, Lally P. Does intrinsic motivation strengthen physical activity habit? Modeling relationships between self-determination, past behaviour, and habit strength. *J Behav Med* 2013;36(5):488-97. doi: 10.1007/s10865-012-9442-0

57. Fitzgerald A, Fitzgerald N, Aherne C. Do peers matter? A review of peer and/or friends' influence on physical activity among American adolescents. *J Adolesc* 2012;35(4):941-58. doi: 10.1016/j.adolescence.2012.01.002

58. Maturo CC, Cunningham SA. Influence of friends on children's physical activity: a review. *Am J Public Health* 2013;103(7):e23-38. doi: 10.2105/ajph.2013.301366

59. Sawka KJ, McCormack GR, Nettel-Aguirre A, et al. Friendship networks and physical activity and sedentary behavior among youth: a systematized review. *The international journal of behavioral nutrition and physical activity* 2013;10:130. doi: 10.1186/1479-5868-10-130

60. Lewis J. Analysing qualitative longitudinal research in evaluations. *Social Policy and Society* 2007;6(4):545-56. doi: 10.1017/S1474746407003880

61. MacPhail A, Gorely T, Kirk D, et al. Children's experiences of fun and enjoyment during a season of sport education. *Research Quarterly for Exercise and Sport* 2008;79(3):344-55.

For peer review only

**Table 1: Characteristics of schools included in the StepSmart Challenge feasibility study**

| | Intervention or Control Group | Single Sex or Co-educational | Free school meal entitlement (%) |
|---|---|---|---|
| **School A** | Control | All Male | 63.7% |
| **School B** | Control | Co-Educational | 7.2% |
| **School C** | Intervention | All Male | 8.0% |
| **School D** | Intervention | All Female | 56.5% |
| **School E** | Intervention | All Female | 54.6% |

For peer review only

**Table 2: Intervention Components and Behaviour Change Techniques (BCTs)**

| **Component** | **Activity/Task** | **Behavioural Change Technique (Michie et al, 2013)** |
|---|---|---|
| Competition | Competition was designed to take place across three levels during Phase one (April – June 2015).<br><br>**1.** School level - £1000 prize for winning school<br>**2.** Team level – trophy for the winning team in each school<br>**3.** Individual level – weekly prizes for highest steps and most improved within each school<br><br>During Phase two (July – Sept 2015) there were individual prizes for the top three participants in each school achieving the highest average number of steps across the 14-week period. This two-phased tapered approach was designed to encourage med-long term PA behaviour change (i.e. extrinsic to intrinsically motivated PA behaviour) | -Set graded tasks<br>-Provide rewards contingent on successful behaviour<br>-Provide feedback on performance |
| Material Rewards/Prizes | Material rewards included coloured stickers, selfie sticks, completion certificates, cinema tickets and £10 sports vouchers. Individual prizes were awarded on a weekly basis under two categories, 'outstanding performance' and 'most improved'. | -Prompt rewards contingent on effort or progress towards behaviour |
| Teams | A team based competition was developed alongside the main school competition to encourage peer support. Ten teams were created within each school (4-5 participants per team). Team captains were selected based on baseline PA data to ensure balance between teams and peer nominations to identify those 'most looked up to'. The highest placed team within each school at the end of Phase One was awarded with a trophy. | -Plan social support/ social change<br>-Facilitate social comparison<br>-Prompt identification as role model/ position advocate |
| Pedometers | Participants were given a Fitbit Zip pedometer and asked to wear throughout every day of the intervention (Phase One and Two). Pedometers provided participants with feedback on daily steps and were | -Goal setting (outcome)<br>-Prompt self-monitoring of behavioural outcome<br>-Provide feedback on performance |

For peer review only

| | | |
|---|---|---|
| | uploaded to the study website via the Fitbit App or using a wireless dongle located at designated areas within schools. | |
| Website | Pedometer data was uploaded to the StepSmart Challenge website and participants could review their daily/weekly scores and view the competition leader board. The website included the provision of motivational messages, weekly challenges and links to other PA resources | -Goal setting (outcome)<br>-Prompt self-monitoring of behavioural outcome<br>-Provide feedback on performance |
| Workbook | A short workbook was given to participants at the start of the intervention. This included 'fun-facts', tips and challenges to promote PA behaviour as well as a section for the participant to record weekly step target (individual and team). | -Provide information on consequences of behaviour in general<br>-Goal setting (outcome)<br>-Prompting generalization of a target behaviour |

For peer review only

**Table 3: Characteristics of focus group participants**

| | | | Average steps per day (measured using accelerometers) | | |
|---|---|---|---|---|---|
| **Participant** | | **Team** | **Baseline** | **Post-intervention** | **12 Month Follow Up** |
| 1 | **School C** | C10 | 9,949 | 8,576 | No valid data |
| 2 | **School C** | C6 | No valid data | No valid data | No valid data |
| 3 | **School C** | C6 | 8,815 | 13,127 | No valid data |
| 4 | **School C** | C7 | 9,325 | 4,099 | 4,099 |
| 5 Winner of 'Most Improved' | **School C** | C1 | 9,264 | 6,687 | 14,246 |
| 6 Winner of 'Walker of the Week' Winner of Summer Competition | **School C** | C5 | 13,326 | 9,563 | 8,039 |
| 1 Winner of 'Walker of the Week' Winner of Summer Competition | **School D** | D2 | 10,940 | 10,684 | 11,784 |
| 2 | **School D** | D9 | 2,787 | No valid data | No valid data |
| 3 Winner of 'Most Improved' | **School D** | D6 | 9,737 | 7,160 | 7,160 |
| 4 | **School D** | D5 | 6,555 | No valid data | 4,088 |
| 5 | **School D** | D5 | 2,782 | No valid data | 5,426 |
| 6 Winner of 'Most Improved' | **School D** | D7 | 9,253 | No valid data | No valid data |
| 1 | **School E** | E7 | 6,495 | 13,080 | 6,129 |
| 2 | **School E** | E7 | 7,330 | No valid data | 9,440 |
| 3 | **School E** | E2 | 6,583 | No valid data | No valid data |
| 4 | **School E** | E9 | 5,915 | No valid data | No valid data |
| 5 Winner of 'Walker of the Week' Winner of Summer Competition | **School E** | E6 | 14,153 | 13,998 | 8,179 |
| 6 Winner of 'Most Improved' | **School E** | E3 | 14,113 | No valid data | 9,988 |
| 7 Winner of 'Walker of the Week' Winner of Summer Competition | **School E** | E3 | 11,330 | No valid data | 5,909 |

* No valid data = Unreturned accelerometer or no valid three-day measurement of data

For peer review only

**Table 4: Overview of the number of participants in (and duration of) each focus group at each time point**

| | **Time points of each focus group** | | | |
|---|---|---|---|---|
| **Intervention schools** | *Baseline (T0)* | *8 weeks (T1)* | *24 weeks (T2)* | *52 weeks (T3)* |
| **School C (All Boys)** | 6 (35 mins) | 6 (21 mins) | 5 (38 mins) | 5 (35 mins) |
| **School D (All Girls)** | 6 (34 mins) | 5 (37 mins) | 6 (40 mins) | 2 (31 mins) |
| **School E (All Girls)** | 7 (36 mins) | 7 (41 mins) | 6 (24 mins) | 7 (24 mins) |

Figure 1: Diagrammatic representation of the temporal thematic pathways that developed during focus group interviews.

Legend: T0 (red), T1 (yellow), T2 (orange) and T3 (green).

For peer review only

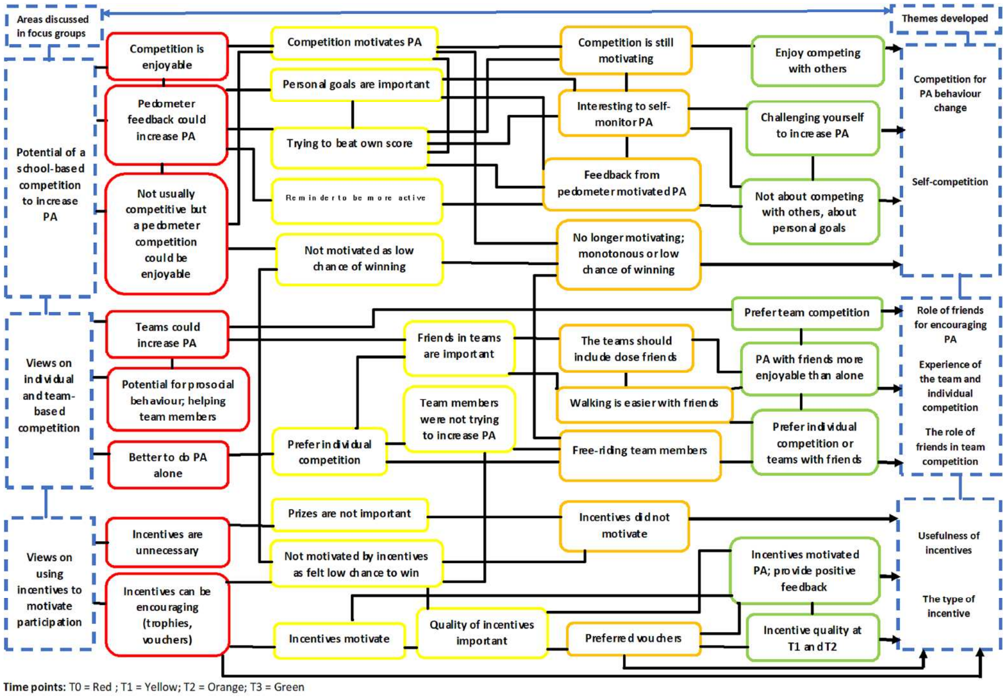


Figure 1: Diagrammatic representation of the temporal thematic pathways that developed during focus group interviews.
Legend: T0 (red), T1 (yellow), T2 (orange) and T3 (green).

592x410mm (300 x 300 DPI)

For peer review only - http://bmjopen.bmj.com/site/about/guidelines.xhtml

**Supplementary Material I: Topic Guide**

| Topic Guide | | | |
|---|---|---|---|
| **Pre-intervention** | **End of phase one** **(External regulation; Introjected regulation)** | **End of phase two** **(Introjected regulation; Identified regulation)** | **12 months post-baseline follow up** **(Integrated motivation)** |
| **Explore the barriers and facilitators to PA**<br><br>What stops you from being physically active?<br>(Relational support)<br><br>Role of parents/friends in your PA? (Autonomy support; Relational support)<br><br>Any ideas to increase PA?<br>(Autonomy support)<br><br>Opportunities for PA within school/community?<br>(Autonomy support; Relational support) | **Explore the experiences of StepSmart**<br><br>How easy is it to find ways in which to be more active?<br>(Autonomy support)<br><br>Did completing the StepSmart Challenge with friends make it easier? Or more pressure?<br>(Autonomy support; Relational support)<br><br>Do you enjoy telling people about your success in terms of PA (e.g. showing others your steps)?<br>(Perceived competence; Relatedness) | **Explore the how participants felt about the different competition elements**<br><br>How did the team competition compare to the individual competition?<br>(Relational support)<br><br>Some of the class were more/less active than you. How did you feel when you compared your steps to the class?<br>(Perceived competence)<br><br>Did team members encourage you to be active or not?<br>(Autonomy support)<br><br>How did you feel if you didn't contribute to the team/school's step count?<br>(Perceived competence) | **Explore if there has been a change in PA (formation of habit)**<br><br>One year on: how active are you now (compared to before you took part)?<br><br>Did you make changes to your PA? Why/why not? *(changes to routine ... active travel, walking with friends etc.)*<br><br>If you did make any changes, which are you still doing? What strategies helped?<br>(Autonomy support) |
| **Explore the acceptability of the intervention components**<br><br>Have you ever heard of…or used a pedometer?<br>What is it you (dis)like about them?<br><br>Feelings on writing down your own daily steps goals in a workbook. | **Explore the attitude towards PA**<br><br>Do you enjoy PA?<br><br>What are your main reasons for being active? (i.e. role of the prizes and other elements?) | **Explore how the StepSmart Challenge instigated any changes in participants PA**<br><br>How do you feel about PA since the StepSmart Challenge?<br><br>What have you enjoyed? | **Explore how participants felt about the StepSmart Challenge**<br><br>Why did you choose to participate?<br><br>Good/not so good aspects of the competition?<br><br>Did the competition motivate – no/at beginning/all throughout? |



For peer review only - http://bmjopen.bmj.com/site/about/guidelines.xhtml

| | | | |
|---|---|---|---|
| (Perceived competence; Autonomy support)<br><br>What features of a website would you find appealing?<br><br>How do you feel about entering a school-based competition?<br><br>Any advantages/disadvantages to team vs individual competitions<br><br>What prizes would motivate young people to take part? | Do you enjoy finding new ways of becoming more active?<br>(Autonomy support)<br><br>Do you think your motivation towards PA has changed? | Has the StepSmart Challenge increased your PA? If not, what would motivate you to be more active?<br><br>Since the StepSmart Challenge, what new opportunities have you found to be active?<br>(Autonomy support)<br><br>Anything outside your control stopping you from being active? (e.g. park proximity, family, other)<br>(Autonomy support; Relational support) | |
| | **To explore the consequences of PA**<br><br>Do you find yourself having more energy?<br><br>Do you feel more confident in being able to achieve things?<br>(Perceived competence)<br><br>How do you feel after you've completed some PA?<br><br>Do you think you are more active now than when you started? Why? | **Explore how participants felt about the use of the pedometer**<br><br>How often did you use the pedometer? What did you like/dislike about it? (e.g. Feedback)<br>(Perceived competence; Autonomy support)<br><br>How many of you are still using the pedometer? Why? | **Explore how participants felt about the team/individual competition**<br><br>Class vs school, was one enjoyed more?<br>(Autonomy support; Relational support)<br><br>Influence of friends on being active?<br>(Autonomy support; Relational support)<br><br>Opportunity to make new friends?<br>(Relational support)<br><br>Feelings about not contributing to the team/school step count?<br>(Perceived competence)<br><br>More motivated if friends wanted to do well in the competition?<br>(Relational support) |



For peer review only - http://bmjopen.bmj.com/site/about/guidelines.xhtml

| | | | |
|---|---|---|---|
| | | **Explore how participants felt about the rewards/prizes**<br><br>What did you think of the prizes?<br><br>Tell me how well they worked to motivate you?<br><br>How did you feel if you didn't win a prize? (Perceived competence)<br><br>If no prizes, but still a competition, would you still be as active? Why? | **Explore how participants felt about the rewards/prizes**<br><br>Type of prizes preferred, including ttrophy or a certificate?<br><br>Not competing against others, but beating your own goal? (Autonomy support; Perceived competence) |
| | | **Explore how the website and Facebook group were used**<br><br>Things you liked/didn't like about the website?<br><br>What did you think of Facebook group? (Autonomy support; Relational support)<br><br>Which way would you prefer to get your information from? (Autonomy support) | **Mood/motivation did these change at different stages of the intervention?**<br><br>Feelings if didn't make as many steps as others? Did that affect your mood? (Perceived competence; Relational support)<br><br>If you did/didn't do well - did that motivate/demotivate you? (Perceived competence; Relational support)<br><br>If felt like don't have a good chance to win would that make you stop trying? (Perceived competence; Relational support) |

Red text illustrates how items in the topic guide are linked the concepts of self-determination theory



For peer review only - http://bmjopen.bmj.com/site/about/guidelines.xhtml

# Consolidated criteria for reporting qualitative studies (COREQ): 32-item checklist

Developed from:
Tong A, Sainsbury P, Craig J. Consolidated criteria for reporting qualitative research (COREQ): a 32-item checklist for interviews and focus groups. *International Journal for Quality in Health Care*. 2007. Volume 19, Number 6: pp. 349 – 357

| No. Item | Guide questions/description |
|---|---|
| **Domain 1: Research team and reflexivity** | |
| *Personal Characteristics* | |
| 1. Inter viewer/facilitator | Which author/s conducted the interview or focus group?<br>**PB, RO'N, RC (page 8)** |
| 2. Credentials | What were the researcher's credentials? E.g. PhD, MD<br>**PhD (PB), PhD (RO'N), MPH (RC) (page 8)** |
| 3. Occupation | What was their occupation at the time of the study?<br>**PB: Research Fellow, UKCRC Centre of Excellence for Public Health/Centre for Public Health**<br>**RO: Research Fellow, UKCRC Centre of Excellence for Public Health/Centre for Public Health**<br>**RC: PhD Student (page 8)** |
| 4. Gender | Was the researcher male or female?<br>**Male (PB), Female (RO'N), Male (RC) (page 8)** |
| 5. Experience and training | What experience or training did the researcher have?<br>**PB: Qualified social worker, training in thematic analysis, Computer Assisted Qualitative Data Analysis (CAQDAS) packages**<br>**RO'N: Experience in the design, conduct and analysis of a photo elicitation and focus group based qualitative research. Training in focus group facilitation and the NVivo**<br>**RC: MPH with a focus on health services research comprising training on qualitative research methods. Formal training in NVivo during PhD (page 8)** |
| *Relationship with participants* | None **(page 8)** |
| 6. Relationship established | Was a relationship established prior to study commencement?<br>**No (page 8)** |
| 7. Participant knowledge of the interviewer | What did the participants know about the researcher? e.g. personal goals, reasons for doing the research |

For peer review only - http://bmjopen.bmj.com/site/about/guidelines.xhtml

| | |
|---|---|
| | **Participants who took part in the focus groups were a sub-sample of those who were involved in the StepSmart intervention study. Participants were told that the purpose of the discussions were to explore their experiences of the StepSmart Project (page 6-7).** |
| 8. Interviewer characteristics | What characteristics were reported about the inter viewer/facilitator? e.g. Bias, assumptions, reasons and interests in the research topic<br>**None** |
| **Domain 2: study design** | |
| *Theoretical framework* | |
| 9. Methodological orientation and Theory | What methodological orientation was stated to underpin the study? e.g. grounded theory, discourse analysis, ethnography, phenomenology, content analysis<br>**The intervention was underpinned by Self-determination theory (page 5). Thematic Analysis Framework Method was used for qualitative data analysis (page 8)** |
| *Participant selection* | |
| 10. Sampling | How were participants selected? e.g. purposive, convenience, consecutive, snowball<br>**Purposive sampling used for participant focus groups (page 6-7)** |
| 11. Method of approach | How were participants approached? e.g. face-to-face, telephone, mail, email<br>**Face-to-face by the teacher (page 7)** |
| 12. Sample size | How many participants were in the study?<br>19 participants<br>**School C (n = 6); School D (n = 6); School E (n = 7) (Table 4)** |
| 13. Non-participation | How many people refused to participate or dropped out? Reasons?<br>**Focus group participants present at each time point varied due to participant absences (mean 6 participants; range 2–7 participants). No participants refused to participate or dropped out. (page 9 and Table 4)** |
| *Setting* | |
| 14. Setting of data collection | Where was the data collected? e.g. home, clinic, workplace<br>**Focus groups were conducted on school premises (page 7)** |
| 15. Presence of non- | Was anyone else present besides the participants and |

| | |
|---|---|
| participants | researchers?<br>**No, only the participants and researchers were present (page 7)** |
| 16. Description of sample | What are the important characteristics of the sample? e.g. demographic data, date<br>**Year 9 classes from 3 schools over 2015-2016**<br>**School C: All male aged between 12 to 14 years**<br>**School D: All female aged between 12 to 14 years**<br>**School E: All female aged between 12 to 14 years**<br>**(page 9 and Table 3)** |
| *Data collection* | |
| 17. Interview guide | Were questions, prompts, guides provided by the authors?<br>**Yes (page 7)**<br>Was it pilot tested?<br>**No (page 7)** |
| 18. Repeat interviews | Were repeat interviews carried out? If yes, how many?<br>**Four were carried out in total. Data were collected one-month pre-intervention (T0); at the end of the team competition (8 weeks) (T1); end of the individual competition (post-intervention) (24 weeks) (T2); and at 12-month follow-up (T3). (page 7)** |
| 19. Audio/visual recording | Did the research use audio or visual recording to collect the data?<br>**Audio recordings were made during each focus group (page 7)** |
| 20. Field notes | Were field notes made during and/or after the interview or focus group?<br>**No** |
| 21. Duration | What was the duration of the interviews or focus group?<br>**Average length of focus groups = 33 minutes (range = 21-41 minutes) (page 9, Table 4)** |
| 22. Data saturation | Was data saturation discussed?<br>**Yes (page 8)** |
| 23. Transcripts returned | Were transcripts returned to participants for comment and/or correction?<br>**Transcripts were not returned to participants for comment, and they did not provided feedback on findings (page 8). However, given the nature of the QLR design core concepts that were apparent at T0 were revisited at subsequent time points to test the validity of the theory.** |

For peer review only

| | |
|---|---|
| **Domain 3: analysis and findings** | |
| *Data analysis* | |
| 24. Number of data coders | How many data coders coded the data?<br>**Three (RC, PB and ME) (page 8)** |
| 25. Description of the coding tree | Did authors provide a description of the coding tree?<br>**Yes (page 8)** |
| 26. Derivation of themes | Were themes identified in advance or derived from the data?<br>**Themes were derived from the data (page 8)** |
| 27. Software | What software, if applicable, was used to manage the data?<br>**NVivo (Version 10, QSR, Southport, UK) (page 8)** |
| 28. Participant checking | Did participants provide feedback on the findings?<br>**Transcripts were not returned to participants for comment, and they did not provided feedback on findings (page 8). However, given the nature of the QLR design core concepts that were apparent at T0 were revisited at subsequent time points to test the validity of the theory.** |
| *Reporting* | |
| 29. Quotations presented | Were participant quotations presented to illustrate the themes/findings? Was each quotation identified? e.g. participant number<br>**Yes (pages 9-15)** |
| 30. Data and findings consistent | Was there consistency between the data presented and the findings?<br>**Yes (pages 9-15)** |
| 31. Clarity of major themes | Were major themes clearly presented in the findings?<br>**Yes (pages 9-15)** |
| 32. Clarity of minor themes | Is there a description of diverse cases or discussion of minor themes?<br>**Sub-themes are discussed (pages 9-15)** |

For peer review only
